# Gastrointestinal-resident, shape-changing microdevices extend drug release in vivo

**DOI:** 10.1126/sciadv.abb4133

**Published:** 2020-10-28

**Authors:** Arijit Ghosh, Ling Li, Liyi Xu, Ranjeet P. Dash, Neha Gupta, Jenny Lam, Qianru Jin, Venkata Akshintala, Gayatri Pahapale, Wangqu Liu, Anjishnu Sarkar, Rana Rais, David H. Gracias, Florin M. Selaru

**Affiliations:** 1 Department of Chemical and Biomolecular Engineering, Johns Hopkins University, Baltimore, MD 21218, USA.; 2 Division of Gastroenterology and Hepatology, Department of Medicine, Johns Hopkins University, Baltimore, MD 21205, USA.; 3 Johns Hopkins Drug Discovery, Baltimore, MD 21205, USA.; 4 Department of Neurology, Johns Hopkins University, Baltimore, MD 21205, USA.; 5 Department of Materials Science and Engineering, Johns Hopkins University, Baltimore, MD 21218, USA.; 6 Department of Chemistry, Johns Hopkins University, Baltimore, MD 21218, USA.

## Abstract

Extended-release gastrointestinal (GI) luminal delivery substantially increases the ease of administration of drugs and consequently the adherence to therapeutic regimens. However, because of clearance by intrinsic GI motility, device gastroretention and extended drug release over a prolonged duration are very challenging. Here, we report that GI parasite–inspired active mechanochemical therapeutic grippers, or theragrippers, can reside within the GI tract of live animals for 24 hours by autonomously latching onto the mucosal tissue. We also observe a notable sixfold increase in the elimination half-life using theragripper-mediated delivery of a model analgesic ketorolac tromethamine. These results provide first-in-class evidence that shape-changing and self-latching microdevices enhance the efficacy of extended drug delivery.

## Teaser

Hookworm inspired, autonomous latching on the gastrointestinal mucosa extends the in vivo retention of a drug delivery device.

## INTRODUCTION

The administration of drugs through the gastrointestinal (GI) tract offers improved compliance over injectables and consequently better treatment outcomes (*1*). Drugs administered via the GI tract are efficiently absorbed into the systemic circulation, in part, due to the enormous intestinal surface area and rich vascularization of the GI tract mucosa (*2*). While the oral route is the more preferred form of drug administration across all age groups, the rectal route is advantageous in the pediatric population as well as during medical emergencies, such as with unconscious patients (*3*, *4*). However, repeated oral or rectal administration often results in imperfect adherence to treatment, a problem that leads to the annual waste of more than 600 billion dollars globally (*1*, *5*). Hence, there is an urgent need to develop orally or rectally administered extended drug delivery systems (*2*, *6*–*8*). For example, mucoadhesive systems, like buccal patches, extend the residence time to 5 or 6 hours, beyond which they are removed due to weak mucosal adhesion (*9*, *10*). Mucus-penetrating particles (MPPs) have shown improved retention compared to mucoadhesive systems, in a mouse model, due to adherence to the mucus layer beneath the outer fast-clearing mucus (*11*, *12*). However, these particles are also removed after a day due to clearance of the underlying mucus layer, and it is unclear at this point if these MPPs can be made resident for longer times. In addition, researchers have reported devices that rely on the variations in density (*13*, *14*) or large size (*15*, *16*) to avoid elimination from the stomach. For example, ring- or star-shaped devices, which are larger than the pylorus opening, can be retained in the stomach for several days before degrading into smaller pieces, which can pass through the pylorus (*8*, *17*). Because these devices are larger than the pylorus opening, which is approximately 2 cm in diameter in adults, the potential risk of gastric obstruction for these devices needs to be evaluated. In addition, the highly acidic gastric fluids in the stomach can react with several drugs delivered by stomach-resident devices, rendering them ineffective.

**Fig. 1:**
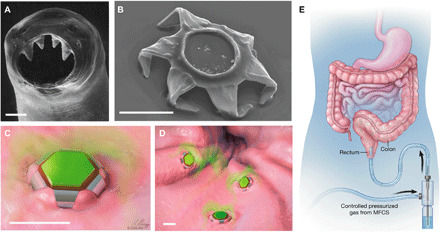
Shape-changing theragrippers as self-latching drug delivery devices. (**A**) Scanning electron microscopy (SEM) image of the ventricular teeth of hookworm *A. duodenale*. The worm uses these sharp teeth to penetrate the mucosa and adheres in the GI tract for up to 2 years. Reprinted from *Human Parasitology*, *4th Ed*. (*18*). Copyright 2013, with permission from Elsevier. (**B**) SEM image of a theragripper in the closed configuration. Like the hookworm, the theragrippers are equipped with sharp microtips. Schematic illustrations of (**C**) a single and (**D**) many theragrippers attached to the mucosal tissue and releasing encapsulated drug (colored in green). Scale bars, 100 μm (A to D). (**E**) Conceptual illustration of the rectal administration of drug-loaded theragrippers using a pressure-actuated microfluidic flow controller. Images (C) to (E) were illustrated by L. Gregg. MFCS, microfluidic flow control system.

Note that the challenge of GI retention has already been solved by some organisms. For example, hookworms such as *Ancylostoma duodenale* (*18*) can reside in the human intestine for up to 2 years. The hookworms insert two pairs of ventricular teeth into tissue, which allows them to securely latch on and resist clearance by GI motility (Fig. 1A). Inspired by these organisms, we developed multiclawed devices with sharp microtips that can latch onto the mucosal tissue. We present the first preclinical evidence that these submillimeter-scale latching tools, called theragrippers, enhance drug release and retention in vivo. In designing these devices, we overcame several engineering challenges. One challenge was the activation of the latching mechanism inside GI lumen in an untethered and autonomous fashion, toward which we used the thermally triggered release of differential stress between metal thin films. Another challenge was to effectively integrate a drug-loaded patch on these microactuators. We investigated delivery of ketorolac as a model drug, which is a widely used pain reliever. We observed that the theragrippers were retained in the colon for 24 hours. This resulted in a significantly higher exposure to the drug, with area under the curve (AUC_last_) almost twice that of pristine drug and a 10-fold higher plasma ketorolac concentration, 12 hours after administration. This proof-of-concept demonstration validates the idea that active, shape-changing, self-latching devices and more specifically theragrippers extend drug release times in the GI tract.

## RESULTS

### Concept and fabrication of theragrippers for rectal drug delivery

We designed theragrippers with multiple sharp microtips (tip diameter, ~1.5 to 3 μm; Fig. 1B), like the hookworm, to ensure effective latching onto the GI mucosa (Fig. 1C). The theragrippers have a combination of thick rigid segments and residually stressed bilayer hinges, capped with a thermosensitive wax layer. They autonomously fold when the wax layer is softened at body temperature, which occurs when thermal equilibrium is reached in the body, and the folding force causes the microtips to penetrate the GI mucosa. While we envision that our devices can function in any part of the GI tract such as the small intestine, colon, or esophagus, in this work, we chose the rectal route of administration, which is the preferred route for pediatric patients and also for localized therapy of diseases like ulcerative colitis. In our approach, the devices are only 250 μm in overall size when open and 150 μm when closed. We deliver thousands of devices that are activated in unison on the colonic mucosa (Fig. 1D) in response to body temperature while releasing the encapsulated drug (Fig. 1E).

**Fig. 2:**
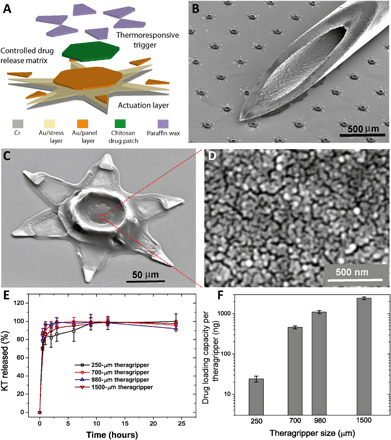
Parallel fabrication of the theragrippers and their in vitro drug loading and release characteristics. (**A**) Functional block diagram illustrating the microfabrication steps for an array of theragrippers, showing the actuation layer, drug-eluting layer, and the thermoresponsive trigger. (**B**) SEM image showing theragrippers next to the tip of a 22-gauge hypodermic needle. The theragrippers are small enough to pass safely through the GI tract without causing any gastric obstruction. (**C**) SEM image showing a single 250 μm, as fabricated theragripper with the drug-encapsulated chitosan patch at the center and the paraffin wax trigger layer on the hinges. (**D**) High-resolution SEM image showing the surface morphology of the chitosan patch at the center of the theragripper. The patch has pores less than 100 nm in size. (**E**) Release characteristics of ketorolac (KT) from theragrippers of four different sizes. (**F**) Plot showing the relative scaling of the drug loading capacity of theragrippers of different sizes. The entire loaded drug gets released over a period of 24 hours. While the 250-μm theragrippers were used for our in vivo experiments in rats, larger 1.5-mm theragrippers can be loaded with about 100 times more drug, for use in larger animal models and humans.

We fabricated the theragrippers using conventional microfabrication techniques with approximately 6000 (250-μm-sized) theragrippers on a single 3-inch wafer substrate (Fig. 2A and fig. S1A). We estimate that over 100,000 theragrippers could be fabricated in parallel on larger 12-inch silicon (Si) wafers that are used in present-day commercial semiconductor foundries. Because the theragripper hinges are very thin (~100 nm), we used 100-μm-scale theragrippers (Fig. 2B and fig. S1, B and C), as smaller theragrippers are more resistant to breakage in vivo. Smaller devices are also less prone to obstruct the GI passage, and the use of multiple devices enhances defect tolerance and spatial distribution of the drug. The theragrippers consist of a metal-polymer hybrid, in which a shape-changing metallic pattern of segments and hinges, made of gold (Au) and chromium (Cr), carries a drug-eluting polymer patch (Fig. 2A) that allows controlled release of drugs. We also incorporated a thin layer (thickness, 8 to 10 μm) of paraffin wax on the hinges of the theragrippers. The wax softens at body temperature and autonomously triggers actuation of the claws inside the animal GI tract (movie S1 and note S1). The materials used to fabricate the theragrippers are biocompatible and do not have any potential toxicity in the small amounts used for drug delivery (note S2).

An important consideration in the design of these devices is the adhesive strength of the drug-loaded polymer layer to the underlying metal scaffold to avoid device failure by delamination of the drug patch due to the strong shear forces encountered during GI peristalsis. We investigated the adhesion of electrodeposited films of charged polymers like chitosan or sodium alginate and drop-casted chitosan films, which have been used previously as matrices for drug delivery (*19*–*21*). We found that, in comparison to alginate and drop-casted chitosan, electrodeposited chitosan had superior adhesion characteristics and could withstand the microfabrication process flow as well as GI peristaltic movements. The dry thickness of the chitosan patch on the theragrippers was 2 to 5 μm (fig. S2, A and B) which was deposited only on the central segment of the theragrippers so that the patch did not impede folding of the claws (Fig. 2C).

### Drug loading and in vitro drug release characteristics of the theragrippers

We were able to tune the porosity of the electrodeposited drug patch, which is important for drug loading and release, by changing the type and extent of deacetylation of chitosan (fig. S2, C to E). The release of fluorescein from chitosan allowed visualization of chemical release over a period of 24 hours (fig. S3 and movie S2). For in vivo experiments, we used ketorolac, a U.S. Food and Drug Administration (FDA)–approved nonsteroidal anti-inflammatory drug (NSAID) used for acute pain management in postoperative patients and patients suffering from rheumatoid arthritis (*22*). Note that ketorolac represents a model drug with a high clearance profile (half-life < 3 hours), and the rapid metabolism and clearance of ketorolac from the body pose a stringent challenge for evaluating extended-release drug delivery systems.

We used medium molecular weight, 85 to 90% deacetylated chitosan, which gave us a nominal pore size of less than 100 nm, and this pore size allowed the release of a substantial amount of ketorolac at an acceptable rate (Fig. 2D). We observed that while coelectrodeposition of ketorolac with chitosan results in a slow release profile (fig. S4), which is attractive for extended drug release, the drug loading capacity per theragripper is very low (1 ng of ketorolac per 250 μm of theragripper). Thus, we adopted a method of drug loading where ketorolac was absorbed in the theragrippers after electrodeposition of chitosan. Note that we found that the conventional process of cross-linking of chitosan with sodium tripolyphosphate after electrodeposition decreased the ability of the film to uptake water and reduced the amount of the drug absorbed in the chitosan matrix (fig. S5). Thus, we soaked the film without cross-linking in the drug solution after electrodeposition. Figure 2E shows the release kinetics of ketorolac from theragrippers of different sizes, where the drug was released over 6 to 9 hours. This method of drug encapsulation (see Materials and Methods) into the theragrippers is highly scalable, and the amount of drug released could be varied by two orders of magnitude, by changing the theragripper size from 250 μm to 1.5 mm (Fig. 2F). Using this method of drug loading, we estimate that each of the 250-μm-sized grippers, weighing approximately 464 ng, carries around 23 ng of ketorolac, which represents an effective drug loading capacity of around 5%. Figure S6 shows the number of theragrippers required to deliver different amounts of drug. For example, 6000 to 7000/kg theragrippers would deliver a therapeutic dosage of 10 mg of ketorolac to a 70-kg adult. The number needed to deliver the same dose goes down substantially for larger 1.5-mm grippers to 70 theragrippers/kg of body weight. We note that even the 1.5-mm grippers are 40 times smaller than the typical human colon diameter of 60 mm. In this study, the in vivo drug delivery experiments were performed in rats, with 250-μm-sized theragrippers. We note that these theragrippers are approximately 32 times smaller than the typical colon diameter of 8 mm in rats used in our experiments, weighing approximately 300 g.

### Force exerted by theragripper claws

One of the critical challenges for successful gastro-retention of the theragrippers in vivo is that they need to generate enough force so that their claws can penetrate through the multiple mucus and epithelial cell layers and through the mucosal barrier. While theragrippers made entirely of polymers (*23*–*25*) are attractive for administration of a larger volume of drug, the moduli and force generated by stimuli responsive polymer actuators are generally lower and hence it is unclear if polymeric theragrippers can generate enough force to penetrate the mucosa (*26*). We used theragrippers with segmented metallic claws composed of stiff metals Cr (modulus ~139 GPa) and Au (modulus ~55 GPa), where the residual stress mismatch (~1 GPa) of the Cr/Au hinges ensures that the claws fold with sufficient force so that the sharp microtips penetrate and latch onto the mucosa autonomously (*27*, *28*). The extent of penetration can be tuned by varying the dimensions of the components of the theragripper and the stress mismatch in the hinge (see note S3) (*29*).

**Fig. 3:**
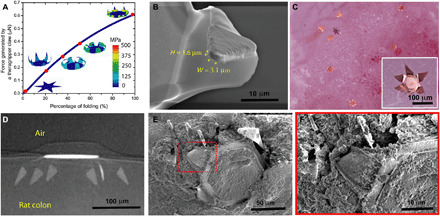
Theragrippers can apply sufficient force to penetrate the mucosa. (**A**) Plot of the force generated by a theragripper as a function of the percentage of folding, generated by FEM. Each claw of the theragripper can generate a maximum force of around 0.6 μN per hinge. Insets show the simulated configurations at different stages of the folding process marked by red dots. The colors in the legend indicate the magnitude of the von Mises stress in the gripper. (**B**) Close-up SEM image of the tip of a theragripper, showing the cross section of the tip having a width (*W*) of approximately 3.1 μm and a height (*H*) of 1.6 μm. To estimate the pressure exerted by this tip as the gripper actuates, we used the Hertz contact mechanics model and assumed the tip to be a sphere of diameter 1.6 to 3.1 μm. (**C**) Ex vivo experiment showing many theragrippers latching onto the colon of a rat. The inset shows the bright-field zoomed-in image of a single theragripper. (**D**) μ-CT image of the cross section of a theragripper penetrating into the colon ex vivo. (**E**) SEM image of a theragripper latching onto the colon mucosa ex vivo. (**F**) Zoomed-in image of the red outlined region in (E), showing the penetration of the claw into the colon tissue.

The small size of the theragripper makes it challenging to directly measure the force. Previously, the force from a similar actuator, albeit with larger dimensions, was measured experimentally (*29*). For the theragrippers used in this study, we used finite element method (FEM) to estimate the force generated by the folding claws (see Materials and Methods). In the simulations, we applied a constraint at the tip of the theragripper, which prevented the tip from folding. A reaction force was generated due to the tendency of folding, and this force was measured in a direction normal to the claw (fig. S7A). The simulations show that the theragripper hinge can generate a force of ~0.5 to 1 μN (Fig. 3A), which can be appreciably tuned by changing the dimensions of the hinge (fig. S7B). We calculated the approximate pressure that each theragripper microtip exerts on the colon using Hertz contact mechanics model and found it to be in the range of 0.4 to 0.6 MPa for a tip diameter of 1.6 to 3.1 μm (see Materials and Methods; Fig. 3B). To compare this pressure to that needed to penetrate the mucosal surface, we performed experiments in which sections of ex vivo rat colons and intestines were penetrated using a 22-gauge hypodermic needle and the force of penetration was measured (fig. S8, A and B). A similar estimation using the Hertz model revealed that a pressure of 0.5 to 1.2 MPa is required for a needle with a tip diameter of 40 to 60 μm to penetrate sections of colon (fig. S8C). Thus, we rationalize that the theragripper microtips, which are more than an order of magnitude smaller than the 22-gauge needle tip, can exert more than enough pressure to break the mucosal barrier.

### Ex vivo and in vivo mucosal attachment of the theragrippers

We performed ex vivo theragripper attachment experiments using rat colon, where the devices were incubated on the mucosal tissue of fresh rat colon at 37°C (Fig. 3C, inset). Micro-computed tomography (μ-CT) of this rat colon shows that the theragripper claws can reach up to 30 to 40 μm into the colon (Fig. 3D). We also visualized and verified the penetration by the theragripper claws using scanning electron microscopy (SEM) (Fig. 3, E and F). It may be noted that the depth of penetration into the colon is strongly controlled by geometric and dimensional factors like the length and layout of the theragripper claws, and we could achieve a depth of penetration of around 300 μm into the colon with theragrippers having a tip to tip size of 1.5 mm (fig. S9).

**Fig. 4:**
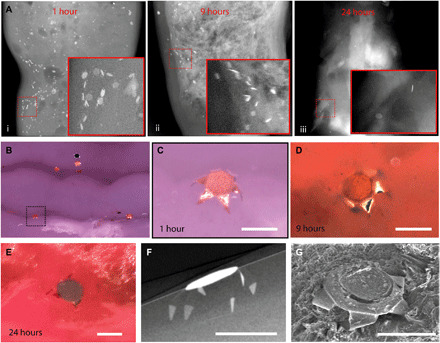
Theragripper attachment to rat colon and retention upon rectal delivery. (**A**) μ-CT images showing the retention of theragrippers in the rat colon (i) 1, (ii) 9, and (iii) 24 hours after rectal administration. The insets show zoomed-in images of the red outlined regions. (**B**) Postmortem optical examination of the rat colon showing attachment of several theragrippers, and (**C**) a zoomed-in image of the section shown by dotted lines in (B). (**D** and **E**) Optical image showing a theragripper attached to the colon (D) 9 and (E) 24 hours after rectal administration. (**F**) Postmortem μ-CT image showing the claws of a theragripper latching into the colon luminal surface in vivo. (**G**) SEM image showing the attachment of the theragripper to the mucosa after rectal administration. Scale bars, 100 μm. (C), (F), and (G) are obtained 1 hour after rectal administration of the theragrippers in the rat.

We then carried out in vivo experiments in rats, in which we deployed thousands of grippers in a single shot using a pneumatic microfluidic controller (PMC). The PMC sends a burst of compressed air (6 to 7 psi) to drive a bolus of liquid with the theragrippers into the colon of the rats (see Materials and Methods). Figure 4A shows CT scan images of sections of extracted rat colon postmortem for theragrippers that were rectally administered in live animals. The images reveal that the theragrippers were retained for at least 24 hours after administration. The colon of the rat appeared normal at the sites of attachment, indicating that there was no short-term gross tissue damage or inflammation over this time period (Fig. 4, B to E, and fig. S10). We also performed μ-CT (Fig. 4F) and SEM (Fig. 4G) on the extracted rat colon tissue, which also verified self-latching of the theragrippers into the colon. Details of these experiments can be found in Materials and Methods.

To demonstrate the applicability of the theragrippers as a resident device in the upper GI tract, we used a porcine model, which allowed endoscopic inspection of the position of the theragrippers in the same animal over time. Around 200 theragrippers were delivered through an endoscope catheter with minimum breakage. Figure S11 (A to C) shows the results of the experiments in porcine stomach and esophagus, which demonstrates the ability of the theragrippers to attach to the mucosa over a duration of 24 hours. Note that while the transit time in the esophagus is less than a minute, the theragrippers remained attached at the specific location for up to a day. We also studied the risks of deploying many grippers in the distal esophagi of pigs, where even after deploying up to 3000 grippers in the esophagus, the animals showed no sign of gastric obstruction or perforation, ate normally, and showed no evidence of pain or distress. Figure S11D shows the magnetic resonance imaging (MRI) image of the whole pig GI tract at 4 weeks after deployment of the grippers, which showed no evidence of the grippers anywhere in the animal. These experiments suggest that the grippers will eventually be safely eliminated from the body by natural mucosal turnover.

### Systemic delivery of a model analgesic by theragrippers

**Fig. 5:**
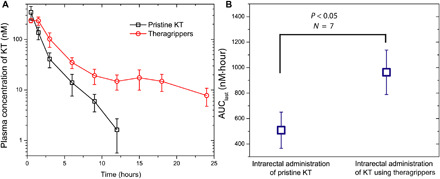
Theragrippers extend the delivery of ketorolac in vivo. (**A**) Plot of the plasma concentration of ketorolac measured in rats as a function of time after pristine or free drug (black) and theragripper (red) were administered intrarectally. **(B)** AUC_last_ comparison for pristine drug and theragripper-formulated drug delivery, showing that the drug exposure increases by approximately twofold with the theragrippers. *P* = 0.0283 for AUC_last_ comparison in (B) is calculated using one-sided Student’s *t* test; *N* = 5 to 7.

We validated in vivo drug release by deploying theragrippers loaded with ketorolac intrarectally, using the pneumatic delivery system mentioned above, to jugular vein cannulated rats. We used 250-μm tip-tip theragrippers, each loaded with approximately 23 ng of ketorolac. Approximately 2000 theragrippers in 1 ml of saline were administered, which translates to an overall administration of 45 μg of ketorolac to each animal in the study (*N* = 7). In a control group of cannulated rats (*N* = 7), we delivered 45 μg of pristine ketorolac dissolved in 1 ml of saline intrarectally using a syringe. Following administration of theragrippers or pristine drug, blood samples were collected via the cannula at 0.5-, 1.5-, 3-, 6-, 9-, 12-, 15-, 18-, and 24-hour time points. We observe that the plasma concentration decreased by 10 times in the first 3 hours (Fig. 5A) for pristine ketorolac, whereas a similar decrease in plasma concentration was seen at 8 hours with theragrippers. Moreover, a sustained exposure was observed from the theragripper formulation between 8 and 18 hours. This indicates an increased half-life and delayed clearance of ketorolac from the theragripper formulation as compared to pristine ketorolac. In addition, the overall increase in exposure is almost twofold (~90%), which suggests a significant increase when compared to pristine ketorolac (Fig. 5B). We observe a notable sixfold increase in half-life from theragrippers (*t*_1/2_ = 12 hours) as compared to *t*_1/2_ = 2 hours following pristine ketorolac dosing (Table 1). Thus, intrarectal administration of theragrippers showed extended and higher exposures of ketorolac compared to pristine drug.

**Table 1:** Ketorolac pharmacokinetic profile following intrarectal delivery in rats. Data expressed as mean ± SEM, *N* = 5 to 7 per group.

	***C*_max_ (nM)**	***T*_max_ (hours)**	***T*_1/2_ (hours)**
Pristine drug	345.7 ± 107.2	0.5	1.94
Theragripper- formulated drug	237 ± 23	0.5	11.83

## DISCUSSION

The adoption of nano- or microfabricated devices including protein encapsulating microparticles, microneedles for transdermal delivery, and microchips containing drug reservoirs has led to considerable progress in localized and extended drug delivery (*30*–*33*). In recent years, dynamic microfabricated smart integrated devices have been developed, which can be controlled by electrical or chemical signals, but these are large centimeter-scale devices (*34*, *35*). Submillimeter-scale miniaturization of dynamic devices is challenging due to the bulkiness of existing batteries and challenges with reduced wireless power coupling to electrically small antennas. Theragrippers overcome this challenge by using energy derived from the triggered release of thin-film differential stress. They operate like a small compressed spring in the form of a thin-film bilayer that stores energy, which can be released when the trigger is dissolved or softened. In our study, the devices are autonomously triggered by physiological temperature. When combined with an appropriately tailored hydrogel patch, theragrippers offer significant tunability and enhancement of drug release characteristics. We note that, other than drug patches, alternate functional modules such as biosensors or microchips could be attached to the theragrippers, opening new possibilities for smart GI-resident diagnostic, monitoring, and therapeutic devices.

Although shape-changing devices have been previously proposed for drug delivery in the GI tract (*36*, *37*) and eye (*38*), they are centimeter sized. The theragrippers, being remarkably smaller in size, by over an order of magnitude, provide a versatile platform for uniform drug dispersion along with applicability for drug release in smaller conduits in the body. In addition, the process is versatile and scalable so that the performance as reported here can be markedly improved by altering the geometry, hydrogel matrix, and drug formulation. For example, one concern is the lack of preferred orientation of the claws of the theragrippers with respect to the mucosal surface, which can be overcome by bidirectional hinge designs (*39*). We have evidence that 250-μm-sized theragripper claws can penetrate 30 to 40 μm into the colon (Figs. 3 and 4), and we observed retention of the grippers in the colon at 24 hours. While our data suggest that the retention time is longer than planar devices in general (*33*), for even longer residence times and drug release in larger animals and eventually humans, theragripper design variables such as claw length, thickness of the metal layers, and the drug patch will need to be further optimized. For example, erosion-based drug release matrices such as thermoplastics show slower release kinetics (*8*) and longer claws can penetrate through thicker mucus layers (fig. S8). We also note that the thermoresponsive nature of the theragrippers makes it essential to use refrigeration for storage, particularly in tropical regions where the air temperature can be higher than 37°C.

Our use of the model drug ketorolac was intended to provide proof of concept, and this concept can be extended to other drugs, as well as to oral administration. We note, however, that rectal administration of ketorolac using theragrippers in itself is also of interest because oral ingestion of ketorolac is known to cause stomach bleeding and ulceration, if taken at higher doses (*40*, *41*). Rectal administration can potentially reduce these complications and partially bypass the first-pass metabolism in the liver. For example, rectal suppositories of similar NSAIDs like Voltaren and Spasmofen have been previously developed and evaluated clinically (*42*, *43*). The increased AUC as achieved by the theragrippers can also reduce side effects and the drug dosage required. In this article, the dosage was kept at 0.15 mg/kg, which can be changed as needed for clinical translation (Fig. 2F and fig. S6).

In terms of a figure of merit, the theragripper formulations of ketorolac showed a half-life of almost 12 hours and residence on colon mucosa measured up to 24 hours. This time is substantially higher than many state-of-the-art GI drug delivery devices such as rectal suppositories. For example, rectal suppositories of ketoprofen (a drug similar to ketorolac) in children aged between 1 and 9 years show a much shorter plasma half-life of 0.6 to 2.5 hours (*44*). The half-life achieved using the theragrippers is also considerably higher than that obtained using oral nano/microparticulate formulations of ketorolac in rats (*45*, *46*). For example, polyethylcyanoacrylate nanoparticles showed a half-life of 2.84 hours following oral gavage, which was only a marginal improvement over the pristine ketorolac solution (*t*_1/2_ = 2 hours). More advanced FDA-approved oral extended-release capsules using osmotic-controlled release oral delivery system or spheroidal oral drug absorption system can maintain plasma concentration of morphine, hydromorphone, or tramadol for 24 hours with elimination half-lives ranging between 8 and 17 hours (*47*, *48*), but these are not GI-resident devices, and hence, their therapeutic window is limited by the duration of normal GI passage after oral delivery.

The concept of physiologically responsive active shape-changing drug delivery systems that provide self-latching in live animals represents a new paradigm in the way drugs can be administered (*49*). Because of their small size, theragrippers can also reach the smaller natural orifices of the body and thus have potential to be applied for other routes of drug administration like nasal, urethral, or vaginal, where sustained drug delivery across the mucus or delivery of capsules is challenging.

## MATERIALS AND METHODS

### Fabrication of the theragrippers

We fabricated the theragrippers by conventional microfabrication techniques (fig. S1A) (*50*, *51*). The main sequential steps of theragripper fabrication are (i) the deposition of a sacrificial layer, (ii) the micropatterning of the differentially stressed bilayer hinges that curve and drive folding and penetration of the theragripper microtips into the mucosa, (iii) the micropatterning of the rigid panels that enhance strength and contain drug-eluting patches, and (iv) the micropatterning of the thermoresponsive trigger that controls stress release and folding of the grippers. First, we deposited a sacrificial layer of copper (300 nm with 15-nm Cr as adhesion layer) on a clean 3-inch silicon wafer using thermal evaporation. We then micropatterned the residual stress bilayer composed of Cr and Au using photolithography with S1813 photoresist (MicroChem Corp.) and thin-film evaporation. We used bilayers with a thicknesses of 50-nm Cr/50-nm Au for the 250-μm grippers and 60-nm Cr/100-nm Au for the 700-μm, 980-μm, and 1.5-mm theragrippers. We defined thicker rigid panels of the theragrippers using a photolithography step with SPR220 photoresist (Megaposit). The rigid panels are composed of electrodeposited Au films with a thickness of 2 to 5 μm. We used an electrodeposition current density of 1 mA/cm^2^ and time of 32 to 80 min at 55° to 60°C using a commercial Au plating solution (Technic).

After electrodeposition of Au, we stripped off the photoresist and patterned the drug patches on the center of the theragrippers using a combination of photolithography and electrodeposition of chitosan. For 250-μm grippers, we used SPR220 photoresist (final thickness, 10 μm) that could accommodate a maximum 2.5-μm-thick chitosan patch after drying. For 700-μm, 980-μm, and 1.5-mm theragrippers, we used AZ9260 photoresist (final thickness, 20 μm) that can accommodate a maximum 5-μm-thick patch after drying of the chitosan. For electrodeposition of chitosan, we used medium molecular weight, 85 to 90% deacetylated chitosan (Sigma-Aldrich). We dissolved the chitosan in an acidic solution of hydrochloric acid and adjusted the final pH to 5. After dissolution, we used a two-stage filtration process using 24- and 2.5-μm pore size filters (Whatman) successively. We carried out the electrodeposition at a current density of 0.2, 0.4, or 0.7 mA/cm^2^ for different durations (fig. S2, A and B), depending on the desired patch thickness. We introduced the dye or drug molecules into the chitosan patch at a later stage, after dissolution of the sacrificial layer and release of the theragrippers from the wafer. Using this approach, we could produce approximately 6000 theragrippers in parallel on a single 3-inch wafer with good reproducibility.

After the deposition of the chitosan patch, the wafer was rested for 3 hours before the photoresist was lifted off in acetone, followed by rinsing in isopropyl alcohol (IPA). We used another step of photolithography to micropattern the thermally responsive trigger layer composed of paraffin wax (melting point, 53° to 58°C; Sigma-Aldrich). We spin-coated molten paraffin wax at 1000 rpm for 10 s and 5000 rpm for 40 s to obtain a uniform film on the wafer. We observed that the thickness of paraffin wax strongly depends on the volume of wax poured on the wafer, the spin coating time, and spin speed/ramp. For the 250-μm theragrippers, we patterned an optimal thickness of around 8 μm on the hinges, which required approximately 0.7 ml of wax on a 3-inch wafer at the spin speeds mentioned above. After spin coating, we allowed the wax to set for 3 to 4 hours and then dissolved the photoresist in acetone and rinsed the wafer using IPA. We released the theragrippers from the silicon wafer substrate using a basic copper etchant BTP (Transene) to ensure the stability of the chitosan film, which otherwise dissolves in conventional acidic copper etchants. Theragripper release from the wafer occurred in 10 to 15 min. Subsequently, we rinsed the theragripper dispersion with deionized (DI) water for at least five times to ensure complete removal of the etchant solution (fig. S1C). The chitosan patches were characterized using a surface profilometer (Dektak) and SEM (JEOL) imaging at 3 to 5 kV.

### In vitro measurements of drug concentrations

For coelectrodeposition of ketorolac and chitosan onto the theragrippers, we added 500 mg of ketorolac to 400 ml of 0.5% (w/w) solution of chitosan and carried out the deposition. For absorption of ketorolac in chitosan-coated theragrippers, we soaked the theragrippers in a ketorolac solution (10 mg/ml) or a fluorescein solution (1 mg/ml) for 36 to 48 hours, which caused the chemicals to get absorbed into the chitosan film. After 48 hours, we thoroughly rinsed the grippers with DI water (at least five to six times by changing the solution) to remove the excess drug or fluorescein and then stored the theragrippers in saline. We measured the ketorolac release from the theragrippers by measuring ultraviolet absorbance at 320-nm wavelength using a spectrophotometer (Molecular Devices). We did triplicate measurements at specific time points by taking out 100 μl of the medium and replacing it by an equal volume of the medium to maintain a proper sink condition. For fluorescein release studies, we prepared the samples in a similar manner and measured the concentrations using fluorescence at 510-nm wavelength in a spectrophotometer.

### FEM to estimate the force generated by the theragripper claws

We simulated the gripper actuation process in Abaqus CAE 6.13 (Dassault Systèmes; the code is available on request from the authors). We used a geometry similar to that used in the experiment, with a hinge, 41 μm wide and 48 μm long. The bilayer hinge consists of Cr (thickness, 50 nm) and Au (thickness, 50 nm). Because of the large aspect ratio between the lateral dimensions and the thickness, we modeled the gripper as a planar shell object and partitioned it to different sections including the center, the hinge, and the tip (fig. S7A). We use linear elastic properties of the materials (table S1) (*52*, *53*). We modeled the Cr/Au bilayer as a composite shell and discretized the mesh using S4R elements (four-node, quadrilateral, doubly curved thin or thick shell with reduced integration). We performed a mesh convergence study to verify mesh independence of the results. We used a static model with consideration of nonlinear geometry and set the boundary conditions as follows, with more details in fig. S7. We fixed the center with zero displacement in *x*, *y*, and *z* directions, and the design was symmetric about the *x* axis. We represented the mismatch strain Δε in the Cr/Au bilayer using the difference between thermal expansion coefficients Δα and a predefined temperature field Δ*T* applied at the hinge, with Δε = Δα • Δ*T*. We considered the stress of the chromium thin film as σ = 1.5 GPa (*54*) and the stress of the neutral Au layer as zero so that Δε = σ_Cr_/*E*_Cr_ • (1 − η_Cr_) = 1.5 GPa/139 GPa × (1 − 0.21) = 0.0085 (*E*_Cr_ = Youngs’ modulus of Cr and η_Cr_ = Poisson’s ratio of Cr) (*55*). We assigned a thermal expansion coefficient of −0.01 (the negative sign is due to the in-plane shrinking of the Cr layer) to the Cr layer and set the temperature field to be Δ*T* = 0.85. We increased the temperature linearly to simulate the process of folding. We constrained the tip in the *z* direction with *dZ* = 0. A reaction force from the tip is generated due to this constraint, and we used this as the force output.

### Estimation of pressure applied by the theragripper and the needle tip

To estimate the pressure exerted by the theragripper microtip and the 22-gauge hypodermic needle tip on the tissue, we used the Hertz contact mechanics model. We approximated the tip of the theragripper as a sphere of diameter 2*R* ≈ 1.6 to 3.1 μm (Fig. 3B) and used the maximum force (*F*) exerted by the theragripper microtip as 0.5 to 1 μN according to the FE simulation. From the Hertz model, we obtained the maximum pressure applied on the colon tissue by the theragripper claw microtip to be $p_{\text{max}}=\frac{1}{\pi }{\left(\frac{6FE^{2}}{R^{2}}\right)}^{1/3}$, where $\frac{1}{E}=\frac{\left(1-\nu_{\text{colon}}^{2}\right)}{E_{\text{colon}}}+\frac{\left(1-\nu_{\text{tip}}^{2}\right)}{E_{\text{tip}}}$ was obtained from the Youngs’ modulus (*E*_colon_ = 0.7 MPa, *E*_tip_ = 55 GPa) and Poisson’s ratio (ν_colon_ ≈ 0.4, ν_tip_ = 0.42) of the colon tissue (*56*) and the Au microtip of the claw. Similarly, for calculations of the pressure applied by the hypodermic needle tip, we assumed the needle tip diameter ≈40 to 60 μm (fig. S8C), and the force exerted to penetrate the colon mucosal surface was obtained from fig. S8B. In addition, the Youngs’ modulus and the Poisson’s ratio of the stainless steel needle tip were assumed to be 200 GPa and 0.3, respectively, and the equations above were used.

### Measurement of the force required to penetrate mucosal tissue

All the experiments in rats were performed according to the protocol RAl9M207 approved by the Animal Care and Use Committee at Johns Hopkins University. We euthanized male Wistar rats (300 g) and removed their colon and small intestine, which were laid flat with the lumen facing up. We immediately carried out tissue stiffness measurements using a tensile tester (Instron). We attached a 22-gauge standard hypodermic needle to a 50 N load cell and advanced the needle toward the tissue sample. We loaded the tissue samples without any tension and only pinned them down just to secure their position. We observed that the needle could penetrate the tissue, and we plotted the displacement and force exerted by the needle (fig. S8A). We observed that different tissue layers of the colon and small intestine required different break pressures. We stopped the tissue penetration experiment when all the tissue layers were completely penetrated by the needle and when we saw the needle coming out of the other end of the tissue. Figure S8B shows the result of 7 to 10 such measurements and compares the superficial penetration and complete perforation of the mucosa.

### Pneumatic delivery of the theragrippers

We used a pneumatic delivery system (Fluigent), in which pressures can be applied selectively using a computer-controlled program (*57*). We stored the grippers in 2-ml vials and then squirted them out with a bolus of liquid at a preset pressure. We conducted several trials to characterize the delivery process. We observed that a pressure greater than 8 psi resulted in a lot of theragripper breakage, while a pressure of 3 to 8 psi could deliver theragrippers with limited breakage. The pneumatic delivery system provided an efficient delivery of the theragrippers in which at least 90% viable theragrippers could be delivered at 6- to 7-psi pressure. We observed a strong dependence of size of the theragripper relative to the tube diameter, on the percentage viability, in which larger theragrippers were more susceptible to breakage at high pressures than smaller theragrippers. It may be noted that, as compared to syringes, the pneumatic system offers a notable improvement in terms of efficiency and ease of use for the delivery of microdevices in a clinical setting.

### μ-CT imaging of theragripper attached to the colon mucosa

During both the ex vivo and in vivo experiments, we identified sections of the colon having attached theragrippers under a microscope and mounted the samples into a computed tomography instrument (RX Solutions). To ensure freshness of the tissue, we imaged the tissue samples within 1 to 2 hours of removal from the animal. We performed the imaging at an x-ray voltage of 80 to 90 kV and a spatial resolution of 1.36 μm. For Figs. 3D and 4F and fig. S9, we tilted the tissue sample to obtain a cross-sectional image.

### SEM imaging of the theragripper attached to rat mucosal tissue

Postmortem, we removed sections of the colon with attached theragrippers and divided them into 1-cm^2^ pieces. We washed the tissue samples in sodium cacodylate buffer and then fixed them in glutaraldehyde for 1 hour. We then stored the samples in sodium cacodylate overnight at 4°C before further processing. We then dehydrated the tissue samples in a graded series of cold (4°C) ethanol (70, 90, and 100%) and hexadimethylsilazane and finally air-dried them by leaving them open to the atmosphere. The samples were then ready for SEM. During SEM (JEOL), we sputtered the tissue samples with 5 nm of Au and an accelerating voltage of 5 kV was used to obtain the images (Figs. 3, E and F, and 4G).

### In vivo adhesion experiments in rat colon and bright-field imaging

We used rats (*N* = 3) to assay the in vivo adhesion characteristics of the theragrippers in the colon. We fasted male Wistar rats (Charles River, MA) weighing approximately 300 g for 1 day to ensure an empty colon before administration of the theragrippers. We then deployed the theragrippers intrarectally under mild anesthesia using isoflurane and oxygen. We inserted a 1.5-mm-diameter medical-grade polytetrafluoroethylene (PTFE) tube (Zeus Inc.), 3 to 4 cm inside the colon of the animal, and then used the delivery system to administer the theragrippers using 6- to 7-psi pressure. We then returned the animals to the cage. At predetermined times after administration, we euthanized the rats and excised the colon. We cleaned the colon with saline and performed the imaging under bright light illumination using an upright microscope (Nikon).

### In vivo adhesion experiments in pig upper GI tract

To demonstrate the ability of theragrippers to adhere to the upper gastric mucosa, we used young farm pigs weighing between 25 and 30 kg. All the experiments in swine model were performed according to protocols SW14M93 and SW17M93 approved by the Animal Care and Use Committee at Johns Hopkins University. The animals were kept on a semi-liquid diet overnight before the start of the experiment. During the experiment, the animals were sedated using an intramuscular dose of a cocktail of ketamine (20 mg/kg) and xylazine (2 mg/kg) and general anesthesia was maintained using isoflurane (1 to 3%) and mechanical ventilation. We intubated the animals and maintained on a dose of saline (0.9%) and lactated Ringer’s (intravenously) throughout the procedure. We then administered the liquid containing the theragrippers using the pneumatic delivery system through a 2.5-mm-diameter medical-grade PTFE catheter that runs through the instrument channel of a standard endoscope (PENTAX Medical Co., Montvale, NJ). For the experiments in the stomach (fig. S11A), we allowed the theragrippers to close for 10 min and then squirted them with water and subjected them to suction through the endoscope. We observed that many grippers could withstand these strong mechanical forces and some of them remained adhered to the stomach wall. Figure S11B (i and ii) shows the images of a specific theragripper before and after the induced flow of water on it. It may be noted that the flow rate of water used (150 ml/min) was much higher than the actual flows that will be encountered by the theragrippers during normal movement of food or water or in normal peristalsis (3 to 5 ml/min). Subsequent experiments were performed in the esophagus of a pig, in which the theragrippers were found to adhere to the mucosal tissue for a prolonged duration of time. Figure S11C (i to iv) shows the time-lapse endoscopic images of a specific location on the esophageal mucosal tissue where the grippers adhered at roughly the same position, demonstrating that theragrippers can remain attached to the esophageal epithelial tissue for 24 hours after administration.

### Safety evaluation in pigs

We assessed the risks of deploying a very high number of grippers by measuring long-term retention in the esophagus. We deployed 3000 grippers in distal esophagus of pigs and followed the animals clinically. Four weeks after the grippers were deployed, the pigs were euthanized and the whole GI tract was removed surgically and imaged with MRI (fig. S11D). To obtain a positive control, we inserted 30 grippers in the proximal esophagus, in between two surgical threads, that prevented them from migrating to the rest of the GI tract. The GI tract from these animals was imaged on a Siemens Magnetom Avanto 3T MRI instrument with a voxel size of 1.0 mm by 1.0 mm by 1.8 mm to search for remnant metal theragrippers in the GI tract.

### Pharmacokinetic studies in rats

We fabricated theragrippers (250 μm) and soaked around 2000 theragrippers in ketorolac (10 mg/ml) solution for 24 hours. We then fasted male Wistar rats, weighing approximately 300 g (Charles River, MA), for 1 day. We removed the theragrippers from the drug solution and washed them at least six times to ensure that no detectable amount of ketorolac was left in the solution. We then delivered the theragrippers inside the rat colon (see above). At predetermined time points, we collected 100 μl of blood via the jugular vein cannula for bioanalysis. We centrifuged the blood samples at 3000 rcf for 10 min at 4°C to separate the plasma and stored the resulting plasma at −80°C, until the bioanalysis was performed. Similarly, we conducted a pharmacokinetic study of pristine ketorolac (45 μg/ml solution in saline) in jugular vein cannulated rats for comparison. We measured ketorolac in the pharmacokinetic samples using liquid chromatography–tandem mass spectrometry. Briefly, we extracted ketorolac from plasma samples following a one-step protein precipitation technique using acetonitrile. We mixed standards, quality controls, and in vivo plasma samples (25 μl) with 100 μl of acetonitrile containing 0.5 μM losartan (internal standard) in low-retention microcentrifuge tubes. We vortexed the mixture for 1 min and centrifuged at 10,000 rpm for 10 min at 4°C. We transferred 50 μl of the supernatant to 250-μl polypropylene autosampler vials, mixed the supernatant with 50 μl of water, and sealed the vials with Teflon caps. We injected a volume of 5 μl onto a Hypersil GOLD C18 analytical column (150 × 2.1 mm inside diameter, 3 μm). We performed chromatographic analysis using an Accela Ultrahigh Performance system consisting of an analytical pump and an autosampler coupled with a TSQ Vantage mass spectrometer (Thermo Fisher Scientific Inc., Waltham, MA). We used a mobile phase composed of 0.1% formic acid in acetonitrile and 0.1% formic acid in H_2_O with gradient elution.

## Supplementary Material

http://advances.sciencemag.org/cgi/content/full/6/44/eabb4133/DC1

Movie S1

Movie S2

Adobe PDF - abb4133_SM.pdf

Gastrointestinal-resident, shape-changing microdevices extend drug release in vivo

## SUPPLEMENTARY MATERIALS

Supplementary material for this article is available at http://advances.sciencemag.org/cgi/content/full/6/44/eabb4133/DC1

## Appendix

View/request a protocol for this paper from *Bio-protocol*.

## References

[1] Traverso G., Langer R., Perspective: Special delivery for the gut. Nature 519, S19 (2015). 25806494 10.1038/519S19a

[2] Garg R., Gupta G. D., Progress in controlled gastroretentive delivery systems. Trop. J. Pharm. Res. 7, 1055–1066 (2008).

[3] Jannin V., Lemagnen G., Gueroult P., Larrouture D., Tuleu C., Rectal route in the 21st Century to treat children. Adv. Drug Deliv. Rev. 73, 34–49 (2014). 24871671 10.1016/j.addr.2014.05.012

[4] Lowry M., Rectal drug administration in adults: How, when, why. Nurs. Times 112, 12–14 (2016). 27071237

[5] Healthprize, “Medication adherence: Pharma’s $637 billion opportunity”; https://healthprize.com/blog/medication-adherence-pharmas-637-billion-opportunity/.

[6] Yun Y. H., Lee B. K., Park K., Controlled drug delivery: Historical perspective for the next generation. J. Control. Release 219, 2–7 (2015). 26456749 10.1016/j.jconrel.2015.10.005 PMC4656096

[7] Tibbitt M. W., Dahlman J. E., Langer R., Emerging frontiers in drug delivery. J. Am. Chem. Soc. 138, 704–717 (2016). 26741786 10.1021/jacs.5b09974

[8] Bellinger A. M., Jafari M., Grant T. M., Zhang S., Slater H. C., Wenger E. A., Mo S., Lee Y. L., Mazdiyasni H., Kogan L., Barman R., Cleveland C., Booth L., Bensel T., Minahan D., Hurowitz H. M., Tai T., Daily J., Nikolic B., Wood L., Eckhoff P. A., Langer R., Traverso G., Oral, ultra–long-lasting drug delivery: Application toward malaria elimination goals. Sci. Transl. Med. 8, 365ra157 (2016. 10.1126/scitranslmed.aag2374 PMC5264553 27856796

[9] Periolia L., Ambrogia V., Angelicia F., Riccia M., Giovagnolia S., Capuccella M., Rossia C., Development of mucoadhesive patches for buccal administration of ibuprofen. J. Control. Release 99, 73–82 (2004). 15342182 10.1016/j.jconrel.2004.06.005

[10] Nafee N. A., Ismail F. A., Boraie N. A., Mortada L. M., Mucoadhesive buccal patches of miconazole nitrate: In vitro/in vivo performance and effect of ageing. Int. J. Pharm. 264, 1–14 (2003). 12972331 10.1016/s0378-5173(03)00371-5

[11] Ensign L. M., Tang B. C., Wang Y.-Y., Tse T. A., Hoen T., Cone R., Hanes J., Mucus-penetrating nanoparticles for vaginal drug delivery protect against herpes simplex virus. Sci. Transl. Med. 4, 138ra79 (2012). 10.1126/scitranslmed.3003453 PMC3817739 22700955

[12] Lai S. K., Wang Y.-Y., Hanes J., Mucus-penetrating nanoparticles for drug and gene delivery to mucosal tissues. Adv. Drug Deliv. Rev. 61, 158–171 (2009). 19133304 10.1016/j.addr.2008.11.002 PMC2667119

[13] Talukder R., Fassihi R., Gastroretentive delivery systems: A mini review. Drug Dev. Ind. Pharm. 30, 1019–1028 (2004). 15595568 10.1081/ddc-200040239

[14] Soppimath K. S., Kulkarni A. R., Rudzinski W. E., Aminabhavi T. M., Microspheres as floating drug-delivery systems to increase gastric retention of drugs. Drug Metab. Rev. 33, 149–160 (2001). 11495501 10.1081/dmr-100104401

[15] Verma M., Vishwanath K., Eweje F., Roxhed N., Grant T., Castaneda M., Steiger C., Mazdiyasni H., Bensel T., Minahan D., Soares V., Salama J. A. F., Lopes A., Hess K., Cleveland C., Fulop D. J., Hayward A., Collins J., Tamang S. M., Hua T., Ikeanyi C., Zeidman G., Mule E., Boominathan S., Popova E., Miller J. B., Bellinger A. M., Collins D., Leibowitz D., Batra S., Ahuja S., Bajiya M., Batra S., Sarin R., Agarwal U., Khaparde S. D., Gupta N. K., Gupta D., Bhatnagar A. K., Chopra K. K., Sharma N., Khanna A., Chowdhury J., Stoner R., Slocum A. H., Cima M. J., Furin J., Langer R., Traverso G., A gastric resident drug delivery system for prolonged gram-level dosing of tuberculosis treatment. Sci. Transl. Med. 11, eaau6267 (2019). 30867322 10.1126/scitranslmed.aau6267 PMC7797620

[16] Liu J., Pang Y., Zhang S., Cleveland C., Yin X., Booth L., Lin J., Lee Y.-A. L., Mazdiyasni H., Saxton S., Kirtane A. R., von Erlach T., Rogner J., Langer R., Traverso G., Triggerable tough hydrogels for gastric resident dosage forms. Nat. Commun. 8, 124 (2017). 28743858 10.1038/s41467-017-00144-z PMC5527117

[17] Zhang S., Bellinger A. M., Glettig D. L., Barman R., Lee Y.-A. L., Zhu J., Cleveland C., Montgomery V. A., Gu L., Nash L. D., Maitland D. J., Langer R., Traverso G., A pH-responsive supramolecular polymer gel as an enteric elastomer for use in gastric devices. Nat. Mater. 14, 1065–1071 (2015). 26213897 10.1038/nmat4355 PMC4772966

[18] B. J. Bogtish, C. E. Carter, T. N. Oeltmann, *Intestinal Nematodes in Human Parasitology* (Elsevier, ed. 4, 2013), pp. 291–327.

[19] Fusco S., Chatzipirpiridis G., Sivaraman K. M., Ergeneman O., Nelson B. J., Pané S., Chitosan electrodeposition for microrobotic drug delivery. Adv. Healthc. Mater. 2, 1037–1044 (2013). 23355508 10.1002/adhm.201200409

[20] Yi H., Wu L.-Q., Bentley W. E., Ghodssi R., Rubloff G. W., Culver J. N., Payne G. F., Biofabrication with chitosan. Biomacromolecules 6, 2881–2894 (2005). 16283704 10.1021/bm050410l

[21] Cheong M., Zhitomirsky I., Electrodeposition of alginic acid and composite films. Colloids Surf. A 328, 73–78 (2008).

[22] Gillis J. C., Brogden R. N., Ketorolac. A reappraisal of its pharmacodynamic and pharmacokinetic properties and therapeutic use in pain management. Drugs 53, 139–188 (1997). 9010653 10.2165/00003495-199753010-00012

[23] Ghosh A., Yoon C., Ongaro F., Scheggi S., Selaru F. M., Misra S., Gracias D. H., Stimuli-responsive soft untethered grippers for drug delivery and robotic surgery. Front. Mech. Eng. 3, 7 (2017). 31516892 10.3389/fmech.2017.00007 PMC6740326

[24] Malachowski K., Breger J., Kwag H. R., Wang M. O., Fisher J. P., Selaru F. M., Gracias D. H., Stimuli-responsive theragrippers for chemomechanical controlled release. Angew. Chem. Int. Ed. Eng. 53, 8045–8049 (2014). 10.1002/anie.201311047 PMC4315180 24634136

[25] He H., Guan J., Lee J. L., An oral delivery device based on self-folding hydrogels. J. Control. Release 110, 339–346 (2006). 16309775 10.1016/j.jconrel.2005.10.017

[26] Breger J. C., Yoon C., Xiao R., Kwag H. R., Wang M. O., Fisher J. P., Nguyen T. D., Gracias D. H., Self-folding thermo-magnetically responsive soft microgrippers. ACS Appl. Mater. Interfaces 7, 3398–3405 (2015). 25594664 10.1021/am508621s PMC4326779

[27] Gultepe E., Randhawa J. S., Kadam S., Yamanaka S., Selaru F. M., Shin E. J., Kalloo A. N., Gracias D. H., Biopsy with thermally responsive untethered microtools. Adv. Mater. 25, 514–519 (2013). 23047708 10.1002/adma.201203348 PMC3832625

[28] Gultepe E., Yamanaka S., Laflin K. E., Kadam S., Shim Y., Olaru A. V., Limketkai B., Khashab M. A., Kalloo A. N., Gracias D. H., Selaru F. M., Biologic tissue sampling with untethered microgrippers. Gastroenterologia 144, 691–693 (2013). 10.1053/j.gastro.2013.01.066 PMC3626272 23399954

[29] Ongaro F., Jin Q., de Cumis U. S., Ghosh A., Denasi A., Gracias D. H., Misra S., Force characterization and analysis of thin film actuators for untethered microdevices. AIP Adv. 9, 055011 (2019).

[30] Sant S., Tao S. L., Fisher O. Z., Xu Q., Peppas N. A., Khademhosseini A., Microfabrication technologies for oral drug delivery. Adv. Drug Deliv. Rev. 64, 496–507 (2012). 22166590 10.1016/j.addr.2011.11.013 PMC3534972

[31] Zhang H., Jackson J. K., Chiao M., Microfabricated drug delivery devices: Design, fabrication, and applications. Adv. Funct. Mater. 27, 1703606 (2017).

[32] Mazzonia C., Tentora F., Strindberg S. A., Nielsen L. H., Keller S. S., Alstrøm T. S., Gundlach C., Müllertz A., Marizza P., Boisen A., From concept to in vivo testing: Microcontainers for oral drug delivery. J. Control. Release 268, 343–351 (2017). 29054373 10.1016/j.jconrel.2017.10.013

[33] Chirra H. D., Shao L., Ciaccio N., Fox C. B., Wade J. M., Ma A., Desai T. A., Planar microdevices for enhanced in vivo retention and oral bioavailability of poorly permeable drugs. Adv. Healthc. Mater. 3, 1648–1654 (2014). 24711341 10.1002/adhm.201300676 PMC4256094

[34] Jiang H., Yu W., Oscai M., Ziaie B., A smart capsule with a hydrogel-based pH-triggered release switch for GI-tract site-specific drug delivery. IEEE Trans. Biomed. Eng. 65, 2808–2813 (2018). 29993401 10.1109/TBME.2018.2818463

[35] Uguz I., Proctor C. M., Curto V. F., Pappa A.-M., Donahue M. J., Ferro M., Owens R. M., Khodagholy D., Inal S., Malliaras G. G., A microfluidic ion pump for in vivo drug delivery. Adv. Mater. 29, 1701217 (2017). 10.1002/adma.201701217 28503731

[36] P. Jarrett, M. J. McGrath, T. S. Jarrett, R. E.-Hayek, A. C. Vanslette, C. A. Rosales, C. D. Blizzard, A. S. Sawhney, Shape changing drug delivery devices and methods. U.S. Patent 10,420,724 (2019).

[37] Babaee S., Pajovic S., Kirtane A. R., Shi J., Caffarel-Salvador E., Hess K., Collins J. E., Tamang S., Wahane A. V., Hayward A. M., Mazdiyasni H., Langer R., Traverso G., Temperature-responsive biometamaterials for gastrointestinal applications. Sci. Transl. Med. 11, eaau8581 (2019). 30996082 10.1126/scitranslmed.aau8581 PMC7797624

[38] Klausner E. A., Lavy E., Stepensky D., Friedman M., Hoffman A., Novel gastroretentive dosage forms: Evaluation of gastroretentivity and its effect on riboflavin absorption in dogs. Pharm. Res. 19, 1516–1523 (2002). 12425470 10.1023/a:1020412817716

[39] Randhawa J. S., Keung M. D., Tyagi P., Gracias D. H., Reversible actuation of microstructures by surface-chemical modification of thin-film bilayers. Adv. Mater. 22, 407–410 (2010). 20217729 10.1002/adma.200902337

[40] Fiedler M. A., Clinical implications of ketorolac for postoperative analgesia. J. Perianesth. Nurs. 12, 426–433 (1997). 9464032 10.1016/s1089-9472(97)90006-x

[41] García Rodríguez L. A., Cattaruzzi C., Troncon M. G., Agostinis L., Risk of hospitalization for upper gastrointestinal tract bleeding associated with ketorolac, other nonsteroidal anti-inflammatory drugs, calcium antagonists, and other antihypertensive drugs. Arch. Intern. Med. 158, 33–39 (1998). 9437376 10.1001/archinte.158.1.33

[42] Novartis, Voltaren® [Package Insert]; https://www.novartis.com.sg/sites/www.novartis.com.sg/files/product-info/1.4.3%20Voltaren%20Supp%20PI%20May%202019.SIN%20%28app%2029%20Jul%202019%29.pdf [accessed 22 June 2020].

[43] Yakoot M., Salem A., Yousef S., Helmy S., Clinical efficacy of Spasmofen® suppository in the emergency treatment of renal colic: A randomized, double-blind, double-dummy comparative trial. Drug Des. Devel. Ther. 8, 405–410 (2014). 10.2147/DDDT.S62571 PMC4018316 24851039

[44] Kokki H., Karvinen M., Suhonen P., Pharmacokinetics of intravenous and rectal ketoprofen in young children. Clin. Pharmacokinet. 42, 373–379 (2003). 12648027 10.2165/00003088-200342040-00005

[45] Radwan M. A., Abou el Ela A. E. S. F., Hassan M. A., El-Maraghy D. A., Pharmacokinetics and analgesic effect of ketorolac floating delivery system. Drug Deliv. 22, 320–327 (2015). 24512312 10.3109/10717544.2014.883189

[46] Radwan M. A., AlQuadeib B. T., Aloudah N. M., Abdul Enein H. Y., Pharmacokinetics of ketorolac loaded to polyethylcyanoacrylate nanoparticles using UPLC MS/MS for its determination in rats. Int. J. Pharm. 397, 173–178 (2010). 20600724 10.1016/j.ijpharm.2010.06.035

[47] Coluzzi F., Mattia C., OROS® hydromorphone in chronic pain management: When drug delivery technology matches clinical needs. Minerva Anestesiol. 76, 1072–1084 (2010). 21102402

[48] Martin C., Baerdemaeker A. D., Poelaert J., Madder A., Hoogenboom R., Ballet S., Controlled release of opioids for improved pain management. Mater. Today 19, 491–502 (2016).

[49] Ghosh A., Xu W., Gupta N., Gracias D. H., Active matter therapeutics. Nano Today 31, 100836 (2020). 32346389 10.1016/j.nantod.2019.100836 PMC7188019

[50] Pandey S., Gultepe E., Gracias D. H., Origami inspired self-assembly of patterned and reconfigurable particles. J. Vis. Exp. 72, e50022 (2013). 10.3791/50022 PMC3600713 23407436

[51] Leong T. G., Randall C. L., Bensona B. R., Bassik N., Stern G. M., Gracias D. H., Tetherless thermobiochemically actuated microgrippers. Proc. Natl. Acad. Sci. U.S.A. 106, 703–708 (2009). 19139411 10.1073/pnas.0807698106 PMC2630075

[52] Kim Y. Y., An advanced characterization method for the elastic modulus of nanoscale thin-films using a high-frequency micromechanical resonator. Materials 10, 806 (2017). 10.3390/ma10070806 PMC5551849 28773165

[53] Espinosa H. D., Prorok B. C., Size effects on the mechanical behavior of gold thin films. J. Mater. Sci. 38, 4125–4128 (2003).

[54] Tyagi P., Bassik N., Leong T. G., Cho J. H., Benson B. R., Gracias D. H., Self-assembly based on chromium/copper bilayers. J. Microelectromech. Syst. 18, 784–791 (2009).

[55] Nikishkov G. P., Curvature estimation for multilayer hinged structures with initial strains. J. Appl. Physiol. 94, 5333 (2003).

[56] Stewart D. C., Rubiano A., Santisteban M. M., Shenoy V., Qi Y., Pepine C. J., Raizada M. K., Simmons C. S., Hypertension-linked mechanical changes of rat gut. Acta Biomater. 45, 296–302 (2016). 27567964 10.1016/j.actbio.2016.08.045 PMC5069177

[57] Choi A., Gultepe E., Gracias D. H., Pneumatic delivery of untethered microgrippers for minimally invasive biopsy. IEEE Int. Conf. Control Autom. 2017, 857–860 (2017). 31456871 10.1109/ICCA.2017.8003172 PMC6711395

[58] Jin Q., Yang Y., Jackson J., Yoon C., Gracias D. H., Untethered single cell grippers for active biopsy. Nano Lett. 20, 5383–5390 (2020). 32463679 10.1021/acs.nanolett.0c01729 PMC7405256

[59] Klokholm E., Berry B. S., Intrinsic stress in evaporated metal films. J. Electrochem. Soc. 115, 823 (1968).

