# Supplementary material for: Gastrointestinal-resident, shape-changing microdevices extend drug release in vivo
Source: Sci Adv. 2020 Oct 28;6(44):eabb4133. doi: 10.1126/sciadv.abb4133 (PMC7608789; doi:10.1126/sciadv.abb4133)
Supplement: http://advances.sciencemag.org/cgi/content/full/6/44/eabb4133/DC1 [file supp_6_44_eabb4133__index.html]

Science Advances | Science AdvancesAAASSearchScience AdvancesMenu

## Supplementary Materials

# Gastrointestinal-resident, shape-changing microdevices extend drug release in vivo

Arijit Ghosh, Ling Li, Liyi Xu, Ranjeet P. Dash, Neha Gupta, Jenny Lam, Qianru Jin, Venkata Akshintala, Gayatri Pahapale, Wangqu Liu, Anjishnu Sarkar, Rana Rais, David H. Gracias, Florin M. Selaru

Download Supplement

**The PDF file includes:**

- Figs. S1 to S11
- Table S1
- Notes S1 to S4
- References

**Other Supplementary Material for this manuscript includes the following:**

- Movie S1
- Movie S2

**Files in this Data Supplement:**

- Adobe PDF - abb4133\_SM.pdf
