# Supplementary material for: Gastrointestinal-resident, shape-changing microdevices extend drug release in vivo
Source: Sci Adv. 2020 Oct 28;6(44):eabb4133. doi: 10.1126/sciadv.abb4133 (PMC7608789; doi:10.1126/sciadv.abb4133)
Supplement: http://advances.sciencemag.org/cgi/content/full/6/44/eabb4133/DC1 [file supp_6_44_eabb4133__1.pdf]

Science Advances
AAAS

advances.sciencemag.org/cgi/content/full/6/44/eabb4133/DC1

Supplementary Materials for

Gastrointestinal-resident, shape-changing microdevices extend drug release in vivo

Arijit Ghosh, Ling Li, Liyi Xu, Ranjeet P. Dash, Neha Gupta, Jenny Lam, Qianru Jin, Venkata Akshintala, Gayatri Pahapale, Wangqu Liu, Anjishnu Sarkar, Rana Rais, David H. Gracias*, Florin M. Selaru*

*Corresponding author. Email: dgracias@jhu.edu (D.H.G.); fselaru1@jhmi.edu (F.M.S.)

Published 28 October 2020, *Sci. Adv.* **6**, eabb4133 (2020)
DOI: 10.1126/sciadv.abb4133

The PDF file includes:

Figs. S1 to S11
Table S1
Notes S1 to S4
References

Other Supplementary Material for this manuscript includes the following:

(available at advances.sciencemag.org/cgi/content/full/6/44/eabb4133/DC1)

Movies S1 and S2

A. Supplementary Figures and Table

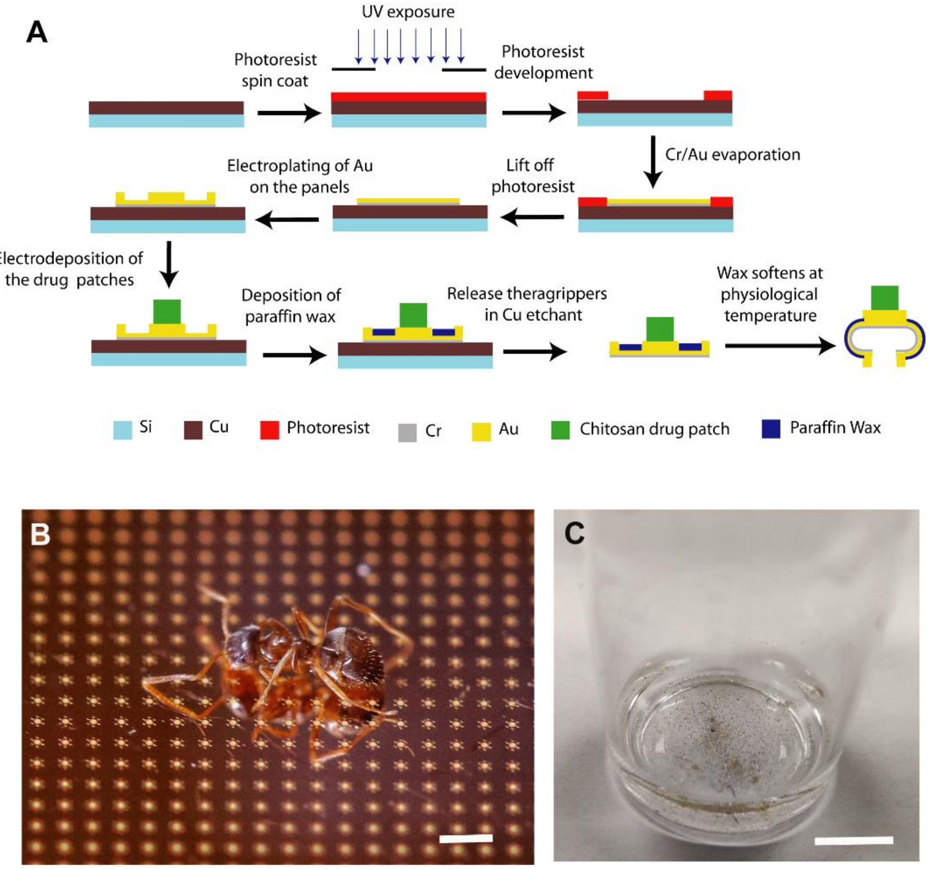

Figure S1: Fabrication process flow of a single residual stress actuator in a theragripper and relative size of the miniaturized theragrippers. (A) Multiple layers are aligned, micropatterned and stacked on the top of each other using photolithographic microfabrication processes. On the dissolution of the sacrificial layer (Cu) in a wet etchant, the theragrippers are released from the wafer. When exposed to the body temperature, the claws fold. **(B)** Photograph of a live fire ant on top of a silicon wafer with as-fabricated theragrippers, illustrating the small size of the theragrippers. Scale bar = 1 mm. *Photo credit :* Wangqu Liu, Johns Hopkins University. **(C)** Photograph of a glass vial with around 6000 theragrippers (250 µm tip-tip) after being released from the wafer, with each theragripper barely visible to the naked eye. Scale bar = 1 cm. *Photo credit :* Arijit Ghosh, Johns Hopkins University.

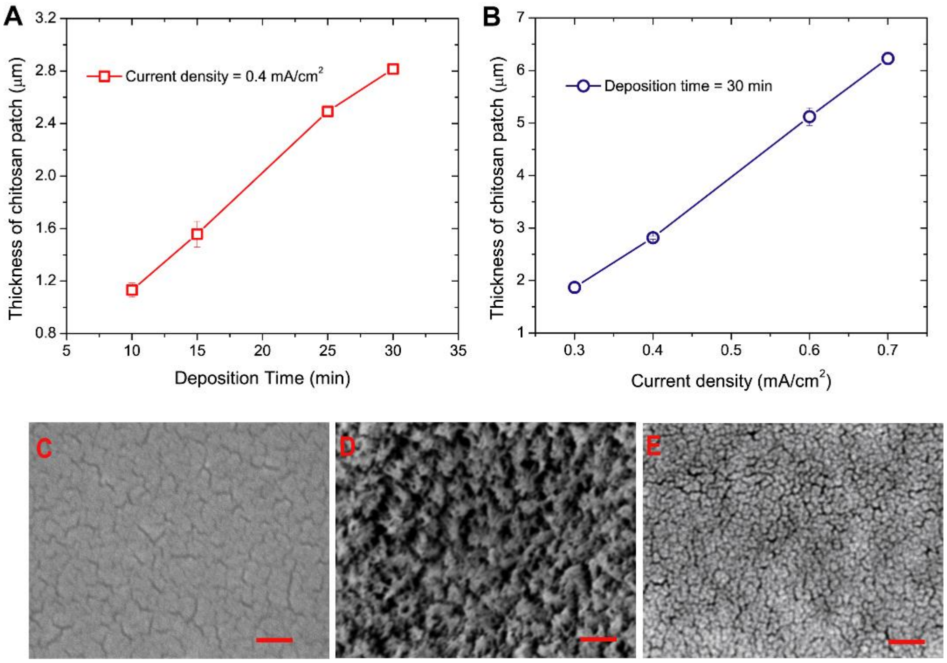

Figure S2: Characterization of the chitosan drug patches. Plot of the thickness of chitosan patches on the theragrippers as a function of, **(A)** deposition time for a fixed current density; **(B)** current density for a fixed deposition time. The results show that the thickness of the patches can be increased by increasing either the deposition time or the current density. In turn, thicker patches can be used to load more drug. **(C-E)** The type and extent of deacetylation of chitosan strongly affects the porosity of the drug patch, which in turn alters drug loading and release characteristics. High resolution SEM images comparing the morphology of the different chitosan films electrodeposited on the theragrippers. **(C)** Chitosan from shrimp shells; **(D)** medium molecular weight chitosan, 75% deacetylated; **(E)** medium molecular weight chitosan, 85 – 90% deacetylated. The amount of ketorolac absorbed by the film in panel C is only 4 – 5 ng per 250 μm theragripper. In comparison, the film in panel D could absorb up to 25 to 30 ng of ketorolac per theragripper, but all the drug was released in less than 1 hour. In this study, we used an optimized chitosan film shown in panel E, which could absorb up to 23 to 24 ng of drug/theragripper, and entirely release it over 7 to 9 hours. All scale bars = 0.5 μm.

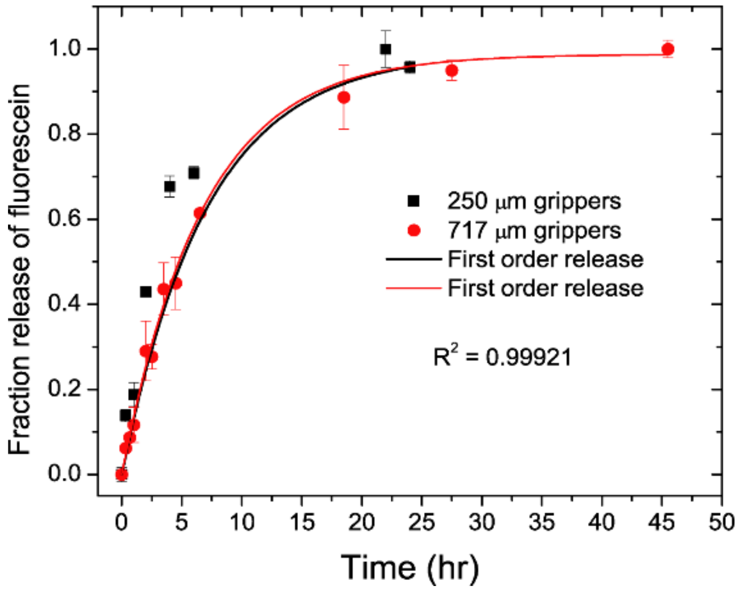

Figure S3: Fractional release of fluorescein from the theragrippers of two different sizes over a period of 24 hours. Fluorescein enabled visualization of the chemical release from theragrippers (Movie S2). The fractional release from both theragripper sizes follows first order kinetics, which indicates a diffusion-based drug release mechanism from the chitosan patch over the time period studied.

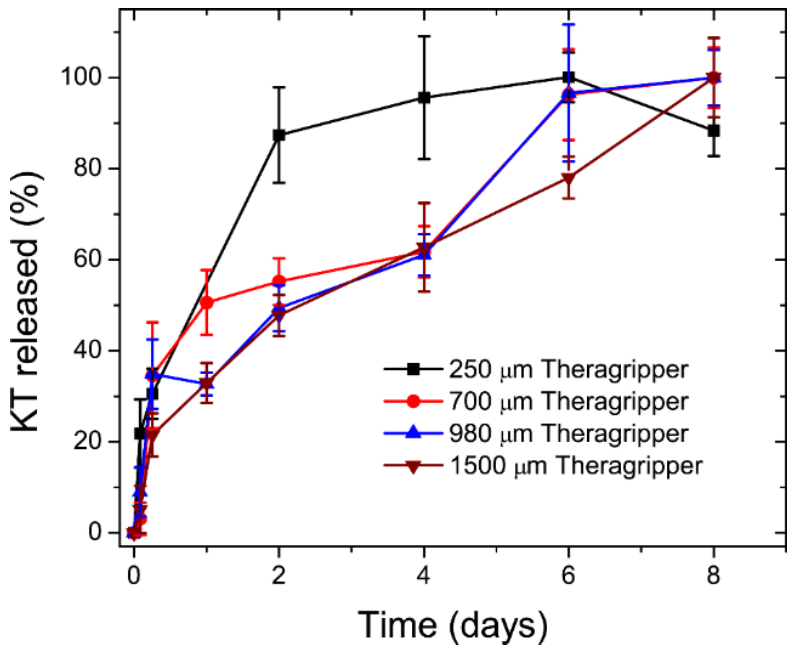

Figure S4: Release kinetics of co-electrodeposited ketorolac from the theragrippers. Percentage release of ketorolac (KT) from theragrippers of different sizes, where the drug was mixed with chitosan during electrodeposition. Though the drug was released slowly over one week, the amount of drug released per theragripper was very low. Plots show mean and standard deviation for three batches of theragrippers.

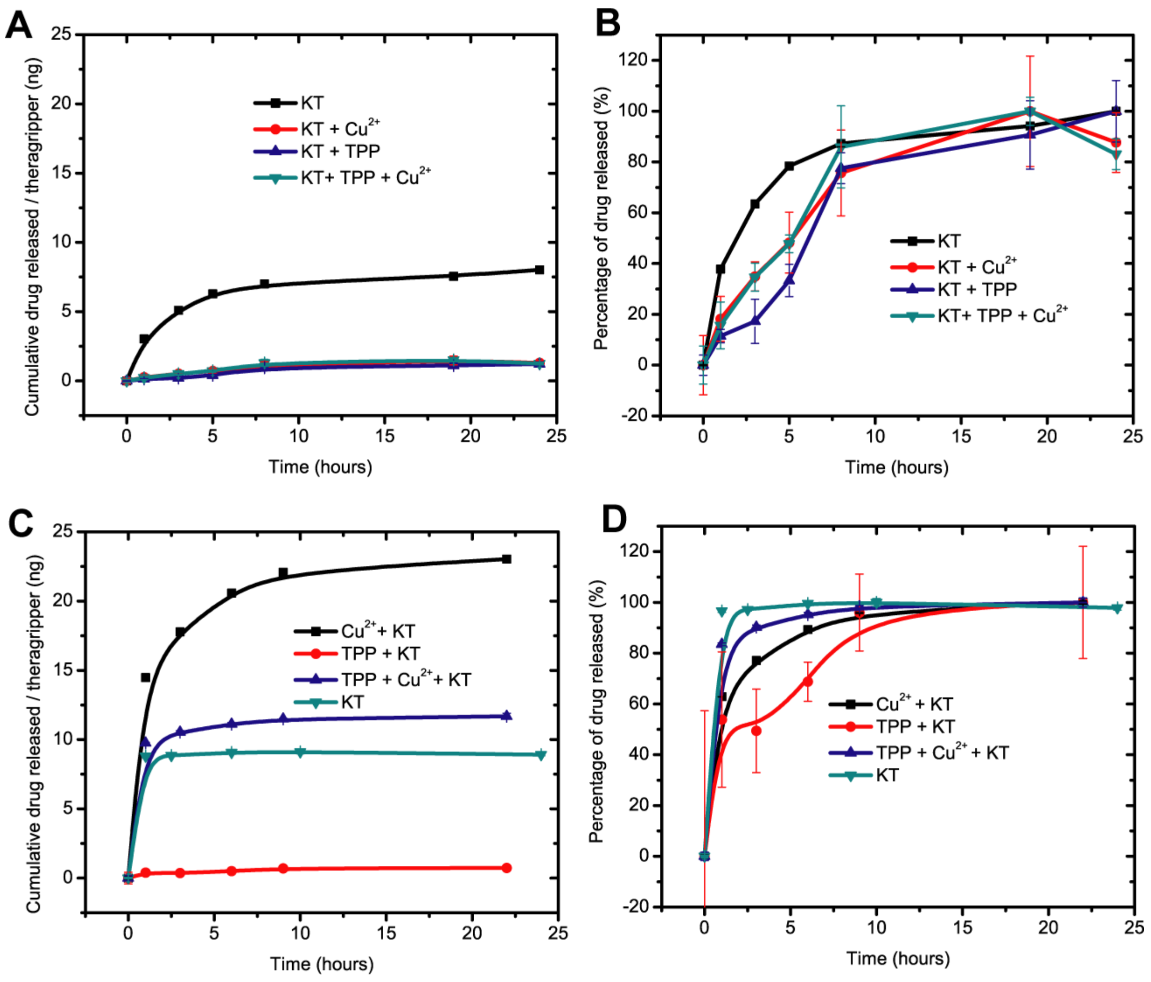

Figure S5: Process optimization of chitosan films to obtain controlled ketorolac release over 24 hours. (A) Cumulative, and **(B)** percentage drug release from chitosan films when the drug was absorbed into the films, immediately after electrodeposition, while the films were still wet. **(C)** Cumulative, and **(D)** percentage drug release from chitosan films when the drug was absorbed in the films after partial drying, to simulate the actual microfabrication process of theragrippers. The treatment with 10% TPP in basic pH, was done for 30 minutes, while the treatment with Cu^{2+} ions was done to simulate the release the theragrippers from the wafer, by soaking the film for 15 minutes in ammoniacal cupric chloride (copper etchant BTP). The legends follow the order of chemical treatments, and the cumulative drug amounts in panels A and C are normalized by the area of a chitosan drug patch on each 250 µm theragripper. The films had the same thickness as the chitosan drug patch on the theragripper. We see that TPP treatment significantly reduces the drug absorption capabilities of the chitosan films for both wet and partially dried films. However, pre-treatment of the films with Cu^{2+} ions increases the drug absorption characteristics and also slows down the drug release profiles significantly. The processing conditions shown in panel C, corresponding to only Cu^{2+} treatment followed by drug absorption (black line) was used for the final experiments, as it yielded a significant drug loading capacity (23 – 24 ng per 250 theragripper) and showed a controlled release over 24 hours.

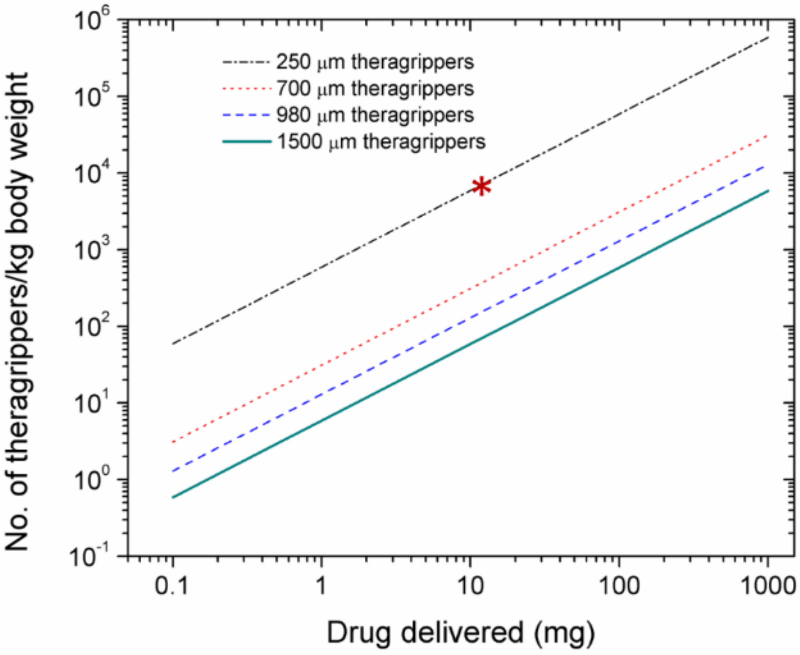

Figure S6: Number of theragrippers needed to deliver different amounts of ketorolac. Theoretical estimates showing the number of theragrippers of different sizes required per kilogram (kg) of body weight, to deliver different amounts of ketorolac, considering a standard human body weight of 70 kg. According to Fig. 2F, we used the following drug capacities for each theragripper, of different sizes: 24 ng (250 µm theragrippers), 459 ng (700 µm theragrippers), 1100 ng (980 µm theragrippers) and 2435 ng (1500 µm theragrippers). The red asterisk corresponds to the dosing used in this paper (0.15 mg/kg of bodyweight), where 250 µm theragrippers could deliver around 10 mg ketorolac, which is a therapeutic dose over a 24-hour period in humans.

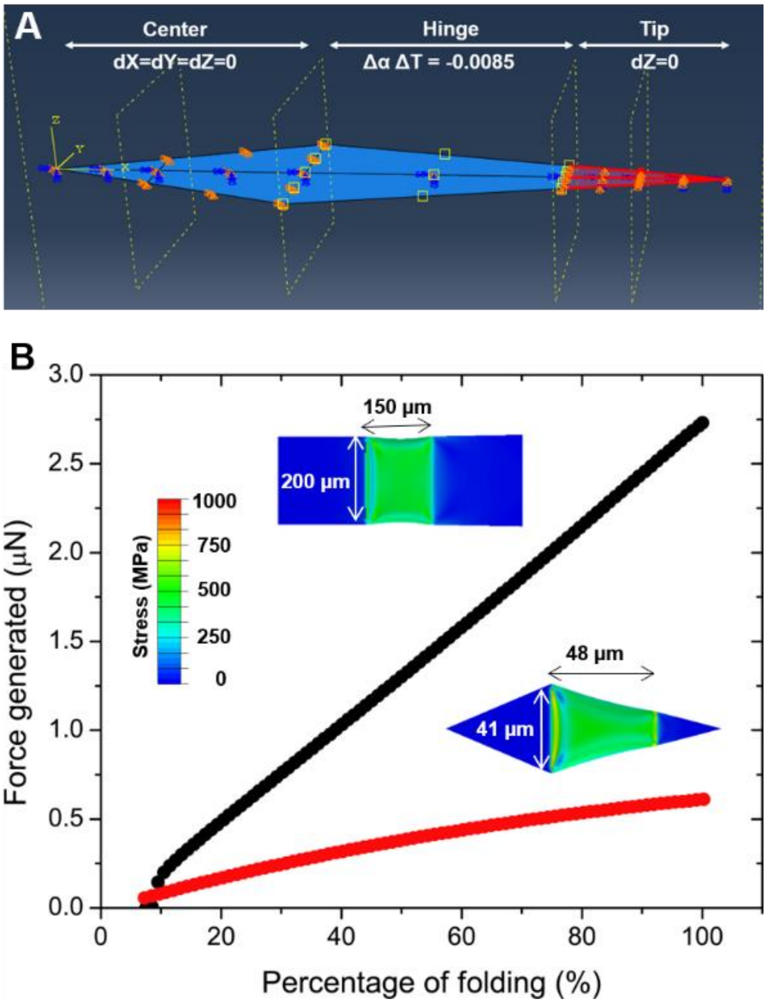

Figure S7: FE simulations used to estimate the force exerted by a theragripper claw. (A) For simplicity, one arm is used in the simulation considering the symmetry of the design. Boundary conditions in FEM are as the following. The *center* of the actuator: displacement in x, y and z directions dX = dY = dZ = 0. The design is symmetric about the x-axis, so for the middle axis, dY = rX = rZ =0. The *hinge*: a predefined temperature field ΔT. The mismatch strain $\Delta\varepsilon$ in the Cr/Au bilayer was represented using the difference between thermal expansion coefficients $\Delta\alpha$ and a predefined temperature field ΔT applied at the hinge, with $\Delta\varepsilon = \Delta\alpha \bullet \Delta T$. The tip (right) was initially allowed to move slightly before mismatch strain reaches 0.0001 and was then constrained in the z direction with dZ = 0. This constraint generated a reaction force from the tip. The reaction force is summarized from all nodes in the tip and used as the force output. **(B)** The force generated from these differentially stressed actuators can be tuned by changing the lateral geometry and size. The red plot shows the force generated by actuators similar to the theragrippers used in this paper (reproduced from Fig. 3A). The black plot shows the force generated by a larger actuator, similar to the ones used in ref. 29. The force obtained from the larger actuator corroborates well with the values of force measured (3 to 5 µN) in ref. 29. The inset color bar shows the stress values in the actuators.

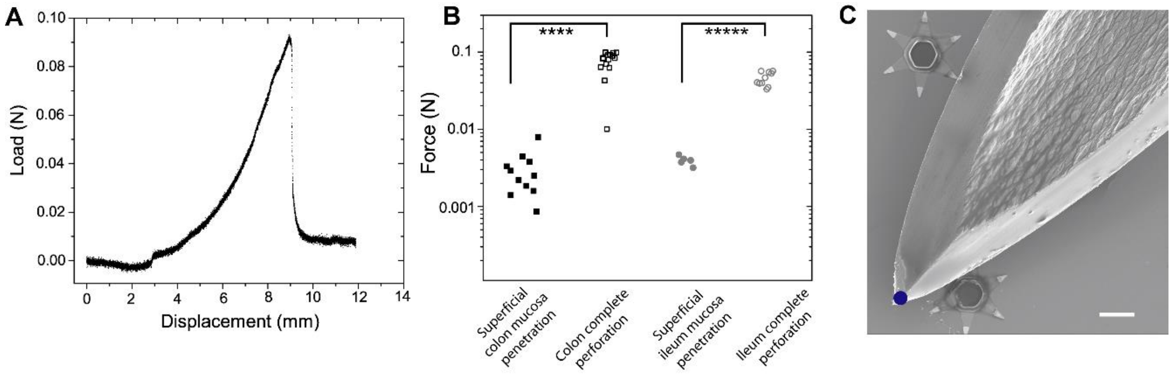

Figure S8: Measurement of force required to penetrate *ex vivo* mucosa in rat colon (large intestine) and ileum (small intestine). (A) A representative load displacement plot obtained during penetration with a 22G needle into parts of *ex vivo* rat GI tract. The drops in the load value correspond to the penetration of the top mucosal layer and the complete perforation of the GI tract respectively. **(B)** Force required to penetrate the top superficial mucosal surface and the complete perforation of *ex vivo* small (ileum) and large intestine (colon) tissue. (**** implies $p < 0.0001$; ***** implies $p < 0.00001$, calculated using one sided students t- test; N = 5 to 12). The force required to penetrate the mucosal layer is significantly higher from the force required to completely perforate the GI tract. **(C)** Close up SEM image of the tip of the hypodermic needle used to do these experiments, with theragrippers in the background, which shows that theragripper microtips are at least one order of magnitude smaller than the hypodermic needle tip. The blue solid circle represents the tip diameter of the needle which was approximately 40 – 60 µm. The force required to penetrate the mucosa with the theragripper microtips is orders of magnitude smaller than that with the hypodermic needle, signifying the higher sharpness of the theragripper microtips. Scale bar = 100 µm.

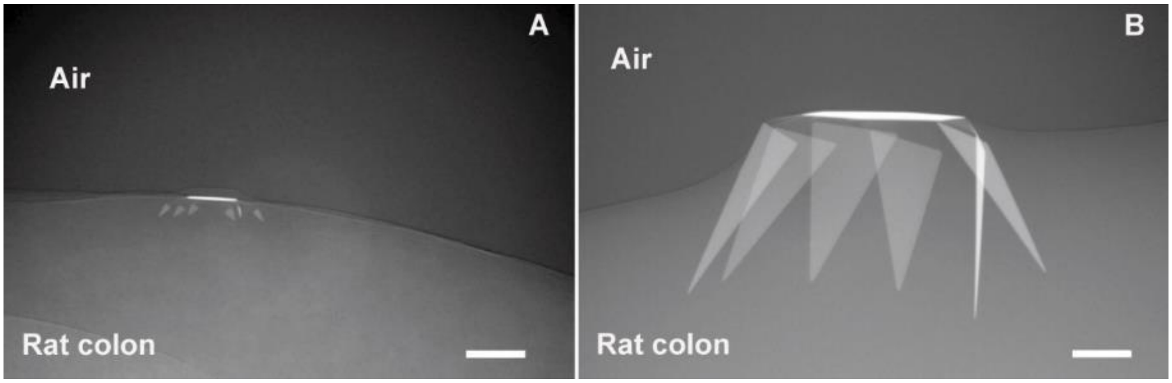

Figure S9: Depth of penetration into the rat colon increases for longer theragripper claws. Micro CT images showing the depth of penetration of theragripper claws into *ex vivo* rat colon for, **(A)** 250 µm tip-to-tip, and **(B)** 1.5 mm tip to tip theragrippers. The depth of penetration increases almost 10 times for theragrippers having a shorter relative hinge length and longer claw lengths. Scale bars = 100 µm.

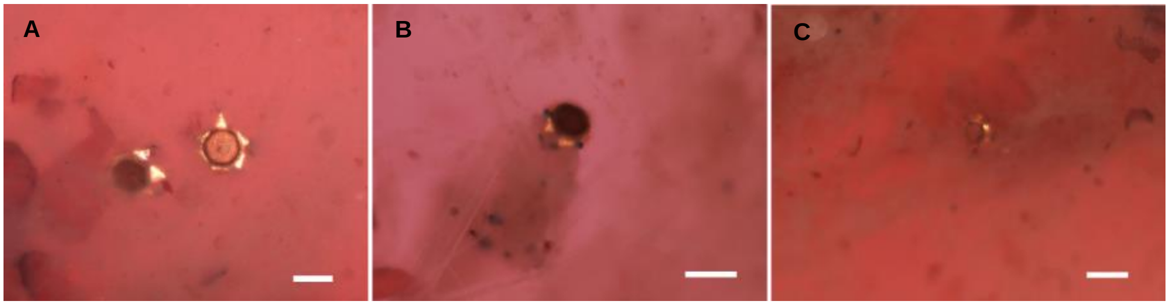

Figure S10: *In vivo* attachment of theragrippers to the rat colon. Optical microscope images showing theragrippers attached to the post-mortem rat colon, **(A)** 1 hour, and **(B-C)** 9 hours after theragripper administration. Scale bars = 100 µm.

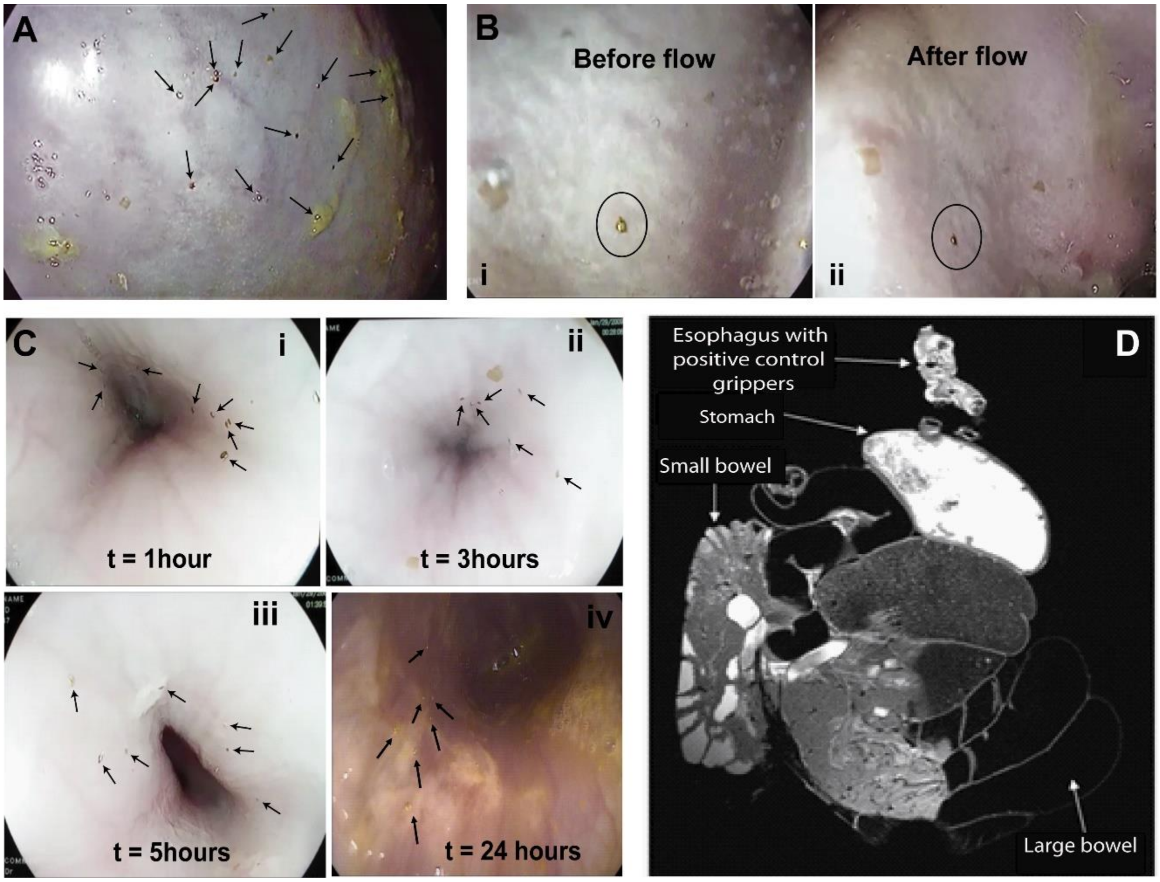

Figure S11: Theragrippers can be retained in the swine upper GI tract and are safely eliminated. (A) A representative image showing theragrippers (717 µm tip-to-tip) adhering to the mucosal tissue in the stomach. The theragrippers were imaged 10 minutes after their administration inside the pig stomach, ensuring that they were closed onto the tissue. (B) Image of a specific theragripper, (i) before, and (ii) after being bombarded with a strong 150 ml/min flow of water using an endoscope. The gripper holds onto the same position onto the mucosal tissue. (C) Time lapse images showing that the theragrippers are latching onto the esophageal tissue (i) 1 hour, (ii) 3 hours, (iii) 5 hours, and (iv) 24 hours after administration. Adhesion experiments in swine upper GI tract performed in two animals. (D) MRI image of pig GI tract 4 weeks after the administration of grippers. Image demonstrating hypodensities in the proximal esophagus (the site where the positive control micrograppers were deployed). No other metallic objects were seen in the rest of the GI tract.

Table S1: Material parameters used to simulate the force exerted by the theragripper claw

Material	Young’s modulus	Poisson’s Ratio
Cr	139 GPa	0.21
Au	55 GPa	0.42

Supplementary Notes

Note S1. Thermoresponsive actuation of the theragrippers: The theragrippers undergo thermoresponsive actuation, in which the paraffin wax trigger layer on the hinges of the gripper softens around 37 °C (*58*) allowing the release of the stored differential stress in the Cr/Au bilayer. The paraffin wax chosen for the theragrippers has a melting point of 53 °C according to manufacturer’s specifications. The claws of the gripper fold in around 10 minutes in water on a Petri dish on a hot plate (Movie S1) or in around 15 minutes on a wet *ex vivo* tissue sample in an oven (preset at 37 °C). The time of actuation can be varied by the rate of heating and by using paraffin wax of different melting points, where a higher melting point will result in a slower actuation of the arms of the gripper. The time of actuation can also be tuned by changing the thickness of the wax layer on the hinges, where a thinner wax layer results in faster actuation. It may be noted that a theragripper without any wax trigger layer actuates almost instantly after dissolution of the sacrificial layer and release of the theragrippers from the wafer.

Apart from the properties of the trigger material as outlined above, inside the animal GI tract, the actuation of the grippers depends on various other factors like the temperature and volume of water used to administer the theragrippers, the convective and thermal flow in the specific location of the body. We have observed that the theragrippers used in this study actuated in around 5-10 minutes when 1 ml of water at room temperature (21 °C) was used to deliver the theragrippers in the rat colon or the pig upper GI tract. Also, the theragrippers used do no actuate prematurely when stored in water at 21 °C. However, theragrippers could prematurely actuate in geographical regions where the air temperature is comparable to or more than 37 °C. In these regions, it will be necessary to implement cold-chain transportation and/or storage of the theragrippers under refrigeration, to ensure proper functionality and avoid premature actuation.

Note S2. Biocompatibility of the theragrippers: The theragrippers are composed of chromium (Cr), gold (Au), paraffin wax and chitosan as the non-therapeutic materials and ketorolac as the therapeutic agent. The materials used for the fabrication of theragrippers are biocompatible at the quantities used. These materials have been used previously in human implants and biomedical devices. Chromium is extensively used as an essential component for tissue engineering applications, is present in many stainless steels and shows no unwanted bodily response. Au is also used regularly in dental implants and nanoparticle mediated drug delivery applications. Paraffin wax and chitosan are commonly used food additives.

The theragrippers used in this study are more than an order of magnitude smaller compared to the nominal GI tract diameter and thus can be safely eliminated after the delivery of drugs. The theragrippers are eliminated by the natural shedding of the gastrointestinal epithelium over a few days. In all our experiments in rodents and pigs, both present and past (*27,28*), we have never observed GI obstruction or any indication of a theragripper-related side effect. In our experiments, the animals ate normally during the experiments and the theragrippers were eliminated from the GI tract over a few days by the natural mucosal turnover. Also, in our experiments we have never seen any adverse toxic effect of the theragrippers on the underlying tissue over a period of 48 hours. We note also that there are many widely practiced gastrointestinal procedures that rely on the use of ingested objects. For example, Sitz markers are used to measure gastrointestinal transit times and hemostasis clips are routinely used to control acute gastrointestinal hemorrhage. Like the theragrippers, these devices are naturally eliminated in stool.

Note S3. Force exerted by the theragripper claws: In order to ensure the successful latching of the theragripper on the GI mucosa, it is important to control the force exerted by the theragripper claws. We modeled the theragripper actuation in this paper using FEM and

estimated the force applied to be approximately 0.5 – 1 μN for 250 μm tip-to-tip theragrippers. The force applied by the grippers depend on several factors like the intrinsic stress mismatch in the actuation bilayer, the material moduli, thickness of the actuator assembly, and the dimensions of the hinge (Fig. S7B). For example, the thickness of the actuator assembly can be used to tune the force for similar Cr/Au actuators (*29*). The stress mismatch is the primary driver of the actuation process and can be tuned over a wide range by using different material combinations. For example, instead of utilizing Cr, one can potentially use iron (Fe) or niobium (Nb) thin films, to form a bilayer with Au with a much higher mismatch (*54, 59*). Also, instead of manipulating only the differential stress of the bilayer, a more effective way is to tune the stress-thickness product of the bilayer actuator, which directly contributes to the resulting radius of curvature or the force obtained from the actuator. The Youngs' modulus of the thin films will directly affect the bendability of the bilayer assembly and will thus affect the force output. Lastly, we note that the lateral geometry of the actuator is important in order to ensure that the primary bending mode is in the desired direction, as the force will be significantly reduced if twisting or buckling modes are present.

Note S4. Mucosal penetration of the theragripper claws: The mobile mucus layer on the mucosal epithelium has different thicknesses in different parts of the GI tract (*12*). Moreover, literature shows that there are two types of mucus layers: an inner strongly adherent layer, which is usually less than 10 μm in thickness and an outer more flowy layer which can be anywhere between 30 – 150 μm in the colon, 100 – 200 μm in the stomach and 10 – 40 μm in the small intestine in humans. The two layers of mucus differ by several hours in terms of times of clearance, and covers the innermost mucosal epithelium, which gets replenished every few days. We also note that the thickness of the mucus layers varies in the GI tract and dependent on several factors including the digestive activity and health.

The retention of the theragripper in the GI tract depends on the depth of penetration of the claws inside the mucus/mucosa. The depth of penetration depends primarily on two factors: (i) The relative length of the theragripper claws with respect to the local mucus thickness, and (ii) the orientation of the theragripper with respect to the mucosal epithelium. The theragrippers are deployed using a pneumatic delivery system which squirts out many grippers in a single shot using a pressure between 6-9 psi. A previous study showed that theragripper adherence increased with increasing pneumatic delivery pressure in *in vitro* models (*57*). Moreover, as we have shown in this paper, using both experiments and finite element modeling, the theragripper claws can exert enough force/pressure to penetrate the mucosal epithelium. Thus, we believe that it is the combination of the pressure applied by the pneumatic delivery system and the pressure applied by the self-folding theragripper claws that allow the theragrippers to latch onto the mucosa. In our study, after deployment on the colon mucus, there is no control of the orientation of the theragrippers with respect to the mucosal epithelium. Consequently, these is a distribution of the grippers having different orientations and at different distances near the mucosal epithelial tissue. However, due to the large number of theragrippers used in our study, a substantial and adequate number of them are oriented towards the epithelium and latch onto the epithelium. As a result, we could find theragrippers in the colon even after 24 hours of administration and the drug delivery could be extended for 24 hours. We anticipate that further improvements in the theragrippers such as designs with longer claws (Fig. S9) and bidirectional foldable hinge designs, as discussed in the main text, could result in more efficient mucosal latching and longer GI retention.

REFERENCES AND NOTES

1. G. Traverso, R. Langer, Perspective: Special delivery for the gut. *Nature* **519**, S19 (2015).
2. R. Garg, G. D. Gupta, Progress in controlled gastroretentive delivery systems. *Trop. J. Pharm. Res.* **7**, 1055–1066 (2008).
3. V. Jannin, G. Lemagnen, P. Gueroult, D. Larrouture, C. Tuleu, Rectal route in the 21st Century to treat children. *Adv. Drug Deliv. Rev.* **73**, 34–49 (2014).
4. M. Lowry, Rectal drug administration in adults: How, when, why. *Nurs. Times* **112**, 12–14 (2016).
5. Healthprize, "Medication adherence: Pharma's $637 billion opportunity"; https://healthprize.com/blog/medication-adherence-pharmas-637-billion-opportunity/.
6. Y. H. Yun, B. K. Lee, K. Park, Controlled drug delivery: Historical perspective for the next generation. *J. Control. Release* **219**, 2–7 (2015).
7. M. W. Tibbitt, J.E. Dahlman, R. Langer, Emerging frontiers in drug delivery. *J. Am. Chem. Soc.* **138**, 704–717 (2016).
8. A. M. Bellinger, M. Jafari, T. M. Grant, S. Zhang, H. C. Slater, E. A. Wenger, S. Mo, Y. L. Lee, H. Mazdiyasni, L. Kogan, R. Barman, C. Cleveland, L. Booth, T. Bensel, D. Minahan, H. M. Hurowitz, T. Tai, J. Daily, B. Nikolic, L. Wood, P. A. Eckhoff, R. Langer, G. Traverso. Oral, ultra–long-lasting drug delivery: Application toward malaria elimination goals. *Sci. Transl. Med.* **8,** 365ra157 (2016).
9. L. Periolia, V. Ambrogia, F. Angelicia, M. Riccia, S. Giovagnolia, M. Capuccella, C. Rossia. Development of mucoadhesive patches for buccal administration of ibuprofen. *J. Control. Release* **99**, 73–82 (2004).
10. N. A. Nafee, F. A. Ismail, N. A. Boraie, L. M. Mortada, Mucoadhesive buccal patches of miconazole nitrate: In vitro/in vivo performance and effect of ageing. *Int. J. Pharm.* **264**, 1–14 (2003).
11. L. M. Ensign, B. C. Tang, Y.-Y. Wang, T. A. Tse, T. Hoen, R. Cone, J. Hanes, Mucus-penetrating nanoparticles for vaginal drug delivery protect against herpes simplex virus. *Sci. Transl. Med.* **4**, 138ra79 (2012).
12. S. K. Lai, Y.-Y Wang, J. Hanes, Mucus-penetrating nanoparticles for drug and gene delivery to mucosal tissues. *Adv. Drug Deliv. Rev.* **61**, 158–171 (2009).
13. R. Talukder, R. Fassihi, Gastroretentive delivery systems: A mini review. *Drug Dev. Ind. Pharm.* **30**, 1019–1028 (2004).
14. K. S. Soppimath, A. R. Kulkarni, W. E. Rudzinski, T. M. Aminabhavi, Microspheres as floating drug-delivery systems to increase gastric retention of drugs. *Drug Metab. Rev.* **33**, 149–160 (2001).
15. M. Verma, K. Vishwanath, F. Eweje, N. Roxhed, T. Grant, M. Castaneda, C. Steiger, H. Mazdiyasni, T. Bensel, D. Minahan, V. Soares, J. A. F. Salama, A. Lopes, K. Hess, C. Cleveland, D.

J. Fulop, A. Hayward, J. Collins, S. M. Tamang, T. Hua, C. Ikeanyi, G. Zeidman, E. Mule, S. Boominathan, E. Popova, J. B. Miller, A. M. Bellinger, D. Collins, D. Leibowitz, S. Batra, S. Ahuja, M. Bajiya, S. Batra, R. Sarin, U. Agarwal, S. D. Khaparde, N. K. Gupta, D. Gupta, A. K. Bhatnagar, K. K. Chopra, N. Sharma, A. Khanna, J. Chowdhury, R. Stoner, A. H. Slocum, M. J. Cima, J. Furin, R. Langer, G. Traverso, A gastric resident drug delivery system for prolonged gram-level dosing of tuberculosis treatment. *Sci. Transl. Med.* **11**, eaau6267 (2019).

16. J. Liu, Y. Pang, S. Zhang, C. Cleveland, X. Yin, L. Booth, J. Lin, Y.-A. L. Lee, H. Mazdiyasni, S. Saxton, A. R. Kirtane, T. von Erlach, J. Rogner, R. Langer, G. Traverso, Triggerable tough hydrogels for gastric resident dosage forms. *Nat. Commun.* **8**, 124 (2017).
17. S. Zhang, A. M. Bellinger, D. L. Glettig, R. Barman, Y.-A. L. Lee, J. Zhu, C. Cleveland, V. A. Montgomery, L. Gu, L. D. Nash, D. J. Maitland, R. Langer, G. Traverso, A pH-responsive supramolecular polymer gel as an enteric elastomer for use in gastric devices. *Nat. Mater.* **14**, 1065–1071 (2015).
18. B. J. Bogtish, C. E. Carter, T. N. Oeltmann, *Intestinal Nematodes in Human Parasitology* (Elsevier, ed. 4, 2013), pp. 291–327.
19. S. Fusco, G. Chatzipirpiridis, K. M. Sivaraman, O. Ergeneman, B. J. Nelson, S. Pané, Chitosan electrodeposition for microrobotic drug delivery. *Adv. Healthc. Mater.* **2**, 1037–1044 (2013).
20. H. Yi, L.-Q. Wu, W. E. Bentley, R. Ghodssi, G. W. Rubloff, J. N. Culver, G. F. Payne, Biofabrication with chitosan. *Biomacromolecules* **6**, 2881–2894 (2005).
21. M. Cheong, I. Zhitomirsky, Electrodeposition of alginic acid and composite films. *Colloids Surf. A* **328**, 73–78 (2008).
22. J. C. Gillis, R. N. Brogden, Ketorolac. A reappraisal of its pharmacodynamic and pharmacokinetic properties and therapeutic use in pain management. *Drugs* **53**, 139–188 (1997).
23. A. Ghosh, C. Yoon, F. Ongaro, S. Scheggi, F. M. Selaru, S. Misra, D. H. Gracias, Stimuli-responsive soft untethered grippers for drug delivery and robotic surgery. *Front. Mech. Eng.* **3**, 7 (2017).
24. K. Malachowski, J. Breger, H. R. Kwag, M. O. Wang, J. P. Fisher, F. M. Selaru, D. H. Gracias. Stimuli-responsive theragrippers for chemomechanical controlled release. *Angew. Chem. Int. Ed. Eng.* **53**, 8045–8049 (2014).
25. H. He, J. Guan, J. L. Lee, An oral delivery device based on self-folding hydrogels. *J. Control. Release* **110**, 339–346 (2006).
26. J. C. Breger, C. Yoon, R. Xiao, H. R. Kwag, M. O. Wang, J. P. Fisher, T. D. Nguyen, D. H. Gracias, Self-folding thermo-magnetically responsive soft microgrippers. *ACS Appl. Mater. Interfaces* **7**, 3398–3405 (2015).

27. E. Gultepe, J. S. Randhawa, S. Kadam, S. Yamanaka, F. M. Selaru, E. J. Shin, A. N. Kalloo, D. H. Gracias, Biopsy with thermally responsive untethered microtools. *Adv. Mater.* **25**, 514–519 (2013).
28. E. Gultepe, S. Yamanaka, K. E. Laflin, S. Kadam, Y. Shim, A. V. Olaru, B. Limketkai, M. A. Khashab, A. N. Kalloo, D. H. Gracias, F. M. Selaru, Biologic tissue sampling with untethered microgrippers. *Gastroenterologia* **144**, 691–693 (2013).
29. F. Ongaro, Q. Jin, U. S. de Cumis, A. Ghosh, A. Denasi, D. H. Gracias, S. Misra Force characterization and analysis of thin film actuators for untethered microdevices. *AIP Adv.* **9**, 055011 (2019).
30. S. Sant, S. L. Tao, O. Z. Fisher, Q. Xu, N. A. Peppas, A. Khademhosseini Microfabrication technologies for oral drug delivery. *Adv. Drug Deliv. Rev.* **64,** 496–507 (2012).
31. H. Zhang, J. K. Jackson, M. Chiao, Microfabricated drug delivery devices: Design, fabrication, and applications. *Adv. Funct. Mater.* **27**, 1703606 (2017).
32. C. Mazzonia, F. Tentora, S. A. Strindberg, L. H. Nielsen, S. S. Keller, T. S. Alstrøm, C. Gundlach, A. Müllertz, P. Marizza, A. Boisen, From concept to in vivo testing: Microcontainers for oral drug delivery. *J. Control. Release* **268**, 343–351 (2017).
33. H. D. Chirra, L. Shao, N. Ciaccio, C. B. Fox, J. M. Wade, A. Ma, T. A. Desai Planar microdevices for enhanced in vivo retention and oral bioavailability of poorly permeable drugs. *Adv. Healthc. Mater.* **3**, 1648–1654 (2014).
34. H. Jiang, W. Yu, M. Oscai, B. Ziaie, A smart capsule with a hydrogel-based pH-triggered release switch for GI-tract site-specific drug delivery. *IEEE Trans. Biomed. Eng.* **65**, 2808–2813 (2018).
35. I. Uguz, C. M. Proctor, V. F. Curto, A.-M. Pappa, M. J. Donahue, M. Ferro, R. M. Owens, D. Khodagholy, S. Inal, G. G. Malliaras, A microfluidic ion pump for in vivo drug delivery. *Adv. Mater.* **29**, 1701217 (2017).
36. P. Jarrett, M. J. McGrath, T. S. Jarrett, R. E.-Hayek, A. C. Vanslette, C. A. Rosales, C. D. Blizzard, A. S. Sawhney, Shape changing drug delivery devices and methods. U.S. Patent 10,420,724 (2019).
37. S. Babaee, S. Pajovic, A. R. Kirtane, J. Shi, E. Caffarel-Salvador, K. Hess, J. E. Collins, S. Tamang, A. V. Wahane, A. M. Hayward, H. Mazdiyasni, R. Langer, G. Traverso. Temperature-responsive biometamaterials for gastrointestinal applications. *Sci. Transl. Med.* **11**, eaau8581 (2019).
38. E. A. Klausner, E. Lavy, D. Stepensky, M. Friedman, A. Hoffman, Novel gastroretentive dosage forms: Evaluation of gastroretentivity and its effect on riboflavin absorption in dogs. *Pharm. Res.* **19**, 1516–1523 (2002).
39. J. S. Randhawa, M. D. Keung, P. Tyagi, D. H. Gracias, Reversible actuation of microstructures by surface-chemical modification of thin-film bilayers. *Adv. Mater.* **22**, 407–410 (2010).

40. M. A. Fiedler, Clinical implications of ketorolac for postoperative analgesia. *J. Perianesth. Nurs.* **12**, 426–433 (1997).
41. L. A. García Rodríguez, C. Cattaruzzi, M. G. Troncon, L. Agostinis, Risk of hospitalization for upper gastrointestinal tract bleeding associated with ketorolac, other nonsteroidal anti-inflammatory drugs, calcium antagonists, and other antihypertensive drugs. *Arch. Intern. Med.* **158**, 33–39 (1998).
42. Novartis, Voltaren® [Package Insert]; https://www.novartis.com.sg/sites/www.novartis.com.sg/files/product-info/1.4.3%20Voltaren%20Supp%20PI%20May%202019.SIN%20%28app%2029%20Jul%202019%29.pdf [accessed 22 June 2020].
43. M. Yakoot, A. Salem, S. Yousef, S. Helmy, Clinical efficacy of Spasmofen® suppository in the emergency treatment of renal colic: A randomized, double-blind, double-dummy comparative trial. *Drug Des. Devel. Ther.* **8**, 405–410 (2014).
44. H. Kokki, M. Karvinen, P. Suhonen, Pharmacokinetics of intravenous and rectal ketoprofen in young children. *Clin. Pharmacokinet.* **42**, 373–379 (2003).
45. M. A. Radwan, A. E. S. F. Abou el Ela, M. A. Hassan, D. A. El-Maraghy, Pharmacokinetics and analgesic effect of ketorolac floating delivery system. *Drug Deliv.* **22**, 320–327 (2015).
46. M. A. Radwan, B. T. AlQuadeib, N. M. Aloudah, H. Y. Abdul Enein, Pharmacokinetics of ketorolac loaded to polyethylcyanoacrylate nanoparticles using UPLC MS/MS for its determination in rats. *Int. J. Pharm.* **397**, 173–178 (2010).
47. F. Coluzzi, C. Mattia, OROS® hydromorphone in chronic pain management: When drug delivery technology matches clinical needs. *Minerva Anestesiol.* **76**, 1072–1084 (2010).
48. C. Martin, A. D. Baerdemaeker, J. Poelaert, A. Madder, R. Hoogenboom, S. Ballet, Controlled release of opioids for improved pain management. *Mater. Today* **19**, 491–502 (2016).
49. A. Ghosh, W. Xu, N. Gupta, D. H. Gracias, Active matter therapeutics. *Nano Today* **31**, 100836 (2020).
50. S. Pandey, E. Gultepe, D. H. Gracias, Origami inspired self-assembly of patterned and reconfigurable particles. *J. Vis. Exp.* **72**, e50022 (2013).
51. T. G. Leong, C. L. Randall, B. R. Bensona, N. Bassik, G. M. Stern, D. H. Gracias Tetherless thermobiochemically actuated microgrippers. *Proc. Natl. Acad. Sci. U.S.A.* **106**, 703–708 (2009).
52. Y. Y. Kim, An advanced characterization method for the elastic modulus of nanoscale thin-films using a high-frequency micromechanical resonator. *Materials* **10**, 806 (2017).
53. H. D. Espinosa, B. C. Prorok, Size effects on the mechanical behavior of gold thin films. *J. Mater. Sci.* **38,** 4125–4128 (2003).

54. P. Tyagi, N. Bassik, T. G. Leong, J. H. Cho, B. R. Benson, D. H. Gracias, Self-assembly based on chromium/copper bilayers. *J. Microelectromech. Syst.* **18**, 784–791 (2009).
55. G. P. Nikishkov, Curvature estimation for multilayer hinged structures with initial strains. *J. Appl. Physiol.* **94**, 5333 (2003).
56. D. C. Stewart, A. Rubiano, M. M. Santisteban, V. Shenoy, Y. Qi, C. J. Pepine, M. K. Raizada, C. S. Simmons, Hypertension-linked mechanical changes of rat gut. *Acta Biomater.* **45**, 296–302 (2016).
57. A. Choi, E. Gultepe, D. H. Gracias, Pneumatic delivery of untethered microgrippers for minimally invasive biopsy. *IEEE Int. Conf. Control Autom.* **2017**, 857–860 (2017).
58. Q. Jin, Y. Yang, J. Jackson, C. Yoon, D. H. Gracias, Untethered single cell grippers for active biopsy. *Nano Lett.* **20**, 5383–5390 (2020).
59. E. Klokholm, B. S. Berry, Intrinsic stress in evaporated metal films. *J. Electrochem. Soc.* **115**, 823 (1968).
